# Coordinate regulation of stress signaling and epigenetic events by Acss2 and HIF-2 in cancer cells

**DOI:** 10.1371/journal.pone.0190241

**Published:** 2017-12-27

**Authors:** Rui Chen, Min Xu, Jason Nagati, Joseph A. Garcia

**Affiliations:** 1 Department of Internal Medicine, University of Texas Southwestern Medical Center, Dallas, Texas, United States of America; 2 Department of Medicine, VA North Texas Health Care System, Dallas, Texas, United States of America; Ludwig-Maximilians-Universitat Munchen Adolf-Butenandt-Institut, GERMANY

## Abstract

Survival of cancer cells in the harsh tumor microenvironment, characterized by oxygen and glucose deprivation, requires rapid initiation of cytoprotective measures. Metabolites whose levels change during stress are ideal signaling cues, particularly if used in post-translational modifications of stress-responsive signal transducers. In cancer cells exposed to oxygen or glucose deprivation, there is an increase in cellular levels of acetate, a substrate for acetate-dependent acetyl CoA synthetase 2 (Acss2) that also stimulates translocation of Acss2 from the cytosol to the nucleus. Nuclear, but not cytosolic, Acss2 promotes acetylation of the stress-responsive Hypoxia Inducible Factor 2α (HIF-2α) subunit by the acetyltransferase/coactivator Creb binding protein (Cbp), a process that facilitates stable Cbp/HIF-2α complex formation. In addition to promoting *de novo* transcription, Cbp and HIF-2α act in concert to regulate local histone 3 epigenetic marks. Exogenous acetate augments Acss2/HIF-2 dependent cancer growth and metastasis in cell culture and mouse models. Thus, an acetate switch in mammals links nutrient intake and stress signaling with tumor growth and metastasis.

## Introduction

The ability to sense and respond to external stress is a requisite property of all living organisms. Diverse environmental stresses encountered *in vivo* oftentimes impinge upon specific genetic regulators to promote cell survival. These genetic regulators, in turn, are frequently controlled by post-translational modifications induced by environmental stress. Linking changes in cellular metabolism to signal transduction via stress-dependent post-translational modifications of genetic regulators allows for a prompt response to environmental stress at the gene expression level.

In metazoans, signal transduction initiated by low oxygen states, or hypoxia, includes *de novo* transcription directed by Hypoxia Inducible Factor (HIF), heterodimeric transcription factors comprised of one of three regulated alpha subunits and a shared beta subunit [1]. HIFs may also respond to other environmental stresses besides hypoxia in a selective manner. For example, HIF-2, but not HIF-1, regulates the cellular response to glucose deprivation [2], which does not activate HIF-1 signaling [3]. In addition to their normal physiological roles, HIF members also play a prominent role in pathophysiological states such as cancer where rapid cellular proliferation tests the limits of oxygen and glucose availability [4].

HIF-1 signaling is largely controlled by HIF-1α protein degradation and coactivator recruitment, regulated by oxygen-dependent prolyl hydroxylases (PHDs) and the asparaginyl hydroxylase Factor Inhibiting HIF-1 (FIH1), respectively [5–7]. However, maximal HIF-2 signaling also requires acetylation and deacetylation of HIF-2α [8–10], which function in a cyclical manner to augment HIF-2 signaling; Cbp-mediated acetylation of HIF-2α facilitates stable Cbp/HIF-2α complex formation that lasts until acetylation is complete, and is reinitiated when Sirtuin 1-mediated HIF-2α deacetylation restores HIF-2α to a naked Cbp substrate [9]. In this study, we link dynamic HIF-2 acetylation and Cbp/HIF-2α complex formation with stress signaling and global epigenetic modifications. We demonstrate the biological relevance of this pathway with cell and mouse cancer models. In the process, we identify a potential causal link of acetate intake with tumor growth and metastasis.

## Materials and methods

### Cell culture

We maintained HT1080 cells (Cat. No. CCL-121, ATCC, Manassas, VA) and HEK293 cells (Cat. No. CRL-1573, ATCC) as previously described with the following modifications[8]. HT1080 cells were grown in either complete (high glucose) medium [Dulbecco’s Modification of Eagle’s Medium (DMEM) with 4.5 g/L (25 mM) glucose, L-glutamine, sodium pyruvate (Cat. No. 10-013-CV, Corning Cellgro, Manassas, VA); 10% heat-inactivated fetal bovine serum (FBS; Cat. No. F4135, Sigma); 1% penicillin/streptomycin (Cat. No. 30-002-CI, Corning Cellgro)] or in low glucose medium [DMEM with L-glutamine (Cat. No. 11966–025, Gibco, Life Technologies, Grand Island NY) supplemented with glucose to final concentration of 1 mM; 10% heat-inactivated FBS; 1% penicillin/streptomycin]. Cells were incubated in either a standard incubator (5% CO_2_, 21% O_2_) or were housed in an incubator located within the hypoxia workstation (5% CO_2_, 1% O_2_). For hypoxia treatments, we prepared extracts within a hypoxia workstation (Coy Laboratories, Grass Lake, MI). For low glucose and acetate treatments, we prepared extracts under normal oxygen conditions. For short chain fatty acid (SCFA) addition, we added sterile acetate to complete medium for a final concentration of 0.5 mM.

### Expression and reporter plasmids

Wild-type (WT) human HIF-2α cDNAs with intact lysine residues (K3) or arginine substitutions (R3: K385R, K685R, K741R) for acetylated lysine residues were previously described [8]. All constructs contain a carboxy terminal hemagglutinin A (HA) tag; where indicated, an amino terminus V5 or S-protein (SP) tag was also present to allow for facile purification as previously described [11]. Site-directed mutagenesis of a parental wild-type mouse Acss2 cDNA was used to generate cDNA encoding amino terminal V5 epitope tagged wild-type (WT), cytosol-restricted (CYT: R668E, K669D), or enzymatic inactive (MUT: T363K) Acss2 as well as WT and CYT Acss2 with an amino SV40 nuclear localization signal tag following the V5 tag (SV40-WT, SV40-CYT). Wild-type (WT) or histone acetyltransferase mutant (HAT: K1540A, F1541A) carboxy terminal c-myc tagged mouse CBP expression constructs were prepared by PCR cloning.

Based upon similar constructs used for efficient expression of shRNA [12] or for efficient knockdown [13], we used pLenti6/V5-GW/lacZ (Invitrogen, Life Technologies, Grand Island, NY) to generate lentiviral (LTV) CMV promoter-driven expression vectors that harbor a DsRed (for knockdown only cell studies) or firefly luciferase (for knockdown mouse flank tumor studies) cDNA in place of the parental lacZ-encoding cDNA, followed by a polylinker containing a concatamer of four different shRNAs for the indicated gene. The control expression cDNA used in the LTV knockdown experiments encodes DsRed (for knockdown only cell studies) or firefly luciferase (for knockdown mouse flank tumor studies) cDNA, followed by a tandem pair of two control shRNAs that generates a concatamer of four shRNAs.

For knockdown/rescue experiments, pLenti6 derivatives were constructed that generate bicistronic cDNA with a human HIF-2α or mouse Acss2 cDNA, which were modified to encode an mRNA resistant to human HIF-2α-selective or human Acss2-selective siRNA or shRNA, placed upstream of an internal ribosome entry site (IRES) and followed by the luciferase-shRNA multimer of interest as described above. The control expression cDNA used in the LTV knockdown/rescue experiments was generated by zipper PCR and produces an Acss2 cDNA with deletions of sequences corresponding to coding exons 3 through 6, which encodes a truncated and non-functional Acss2 protein.

### Lentivirus stable transduction

Lentiviruses were generated by co-transfection of the expression vector of interest with packaging plasmids psPAX2 and pMD2G. The day before transduction, HT1080 cells were trypsinized and 2 x 10^5^ cells per well plated in 1 mL complete culture medium in a 6-well plate for overnight incubation at 37°C. On the day of transduction, media was removed and replaced with 1 ml of complete medium with 10 μg/ml polybrene (Cat. No. 107689, Sigma, St. Louis, MO). Lentiviral particles were thawed to room temperature, mixed gently, and added to the HT1080 cells. After gently swirling to mix, cells were incubated overnight. After 12 hr, culture medium was replaced with 2 ml of complete medium containing 10 μg/ml blasticidin S (Cat. No. ant-bl, Invivogen, San Diego, CA), which was replaced every 2 days until one week after all control cells had died. Positive cells were propagated in 1 μg/ml blasticidin S for two weeks, and then were frozen down. For experiments, cells were thawed and allowed to grow for three passages before use.

### Immunodetection

Proteins were analyzed by immunoblotting with primary antibodies for the following antigens: human p300 (1:500 dilution; Cat. No. sc-584, Santa Cruz Biotechnology, Santa Cruz, CA), human CBP (1:500 dilution; Cat. No. 4772, Cell Signaling Technology, Danvers, MA), human Acss2 (1:500 dilution; Cat. No. ab66038, Abcam), human HIF-2α (1:1,000 dilution; Cat. No. NB100-132, Novus Biologicals, Littleton, CO), TATA-binding protein (TBP) (1:1,000 dilution; Cat. No. sc-204, Santa Cruz Biotechnology), α-tubulin (1:10,000 dilution; Cat. No. T9026, Sigma), HA (1:5,000 dilution; Cat. No. H9658, Sigma), V5 (1:5,000 dilution; Cat. No. R960-25, Life Technologies), or c-Myc (1:5,000 dilution; Cat. No. sc-789, Santa Cruz Biotechnology).

### Ectopic HIF-2α acetylation

HT1080 cells expressing amino-terminal S-peptide (SP) epitope tagged and carboxy-terminal hemagglutinin A (HA) epitope tagged wild-type (WT) HIF-2α were pretreated with sirtinol (Cat. No. 510 8474, Chembridge Corporation, San Diego, CA; 5 μM final concentration) and nicotinamide (NAM) (Cat. No. N0636, Sigma; 10 mM final concentration) for 6 hr prior to initiation of stress or to acetate addition. Ectopic HIF-2α was purified using SP-agarose from whole cell extracts, prepared with a kit (Cat. No. 40010, Active Motif, Carlsbad, CA) supplemented with 1× protease inhibitor cocktail (Cat. No. P8340, Sigma), 1 mM PMSF, 10 mM NAM, and 5 μM sirtinol, and subjected to immunoblot analyses as previously described[8]. Similarly, amino-terminal V5-peptide (V5) epitope tagged wild-type K3 or mutant R3 (K385R, K685R, K741R) HIF-2α expressed in HT1080 cells was purified using V5-agarose before immunoblot analysis.

### Endogenous HIF-2α acetylation

HT1080 cells in a single 100 mm plate for each time-point were pretreated for 6 hr in complete medium with 5 μM sirtinol and 10 mM NAM, and then cultured in the appropriate medium under the indicated conditions. Whole cell extracts were prepared using a kit (Cat. No. 40010, Active Motif) supplemented with 1× protease inhibitor cocktail, 1 mM PMSF, 10 mM NAM, and 5 μM sirtinol. Endogenous HIF-2α was incubated with a monoclonal human HIF-2α antibody (Cat. No. NB100-132, Novus Biologicals) for 1 hr and then was immunoprecipitated using protein G magnetic beads (Cat. No. 54002, Active Motif). Aliquots were immunoblotted for endogenous HIF-2α or acetyl lysine as described [9].

### Endogenous protein lysine acetylation

Stable HT1080 cells expressing shRNA directed against human Acss2 or expressing control non-targeting shRNA [14] maintained under blasticidin S selection (1 μg/mL medium) were plated in a single 100 mm plate and grown until 90% confluency, then were changed to the appropriate medium without blasticidin S for growth under the indicated conditions. Cells were treated for the last 6 hr of the treatment period with 5 μM sirtinol and 10 mM NAM. Whole cell extracts were prepared using a kit (Cat. No. 40010, Active Motif) supplemented with 1× protease inhibitor cocktail, 1 mM PMSF, 10 mM NAM, and 5 μM sirtinol. Aliquots (15 μg) of whole cell extracts were electrophoresed on a 10% PAGE gel, transferred to a PVDF membrane (Cat. No. IPVH00010, EMD Millipore), and immunoblotted with antibody recognizing acetylated lysine residues (1:1,000 dilution; Cat. No. 9441, Cell Signaling Technology) after pre-blocking. Parallel samples were analyzed by immunoblotting to detect endogenous human Acss2 (1:1,00 dilution; Cat. No. D19C6, Abcam) and α-tubulin (1:5,000 dilution; Cat. No. T9026, Sigma).

### Endogenous protein immunoprecipitation

Immunoprecipitation of endogenous proteins was accomplished using a Universal Co-IP kit (Cat. No. 54002, Active Motif). HT1080 nuclear extracts were first incubated with protein A agarose beads. Cleared supernatants were then incubated with HIF-2α antibody (Cat. No. NB100-132, Novus Biologicals) or normal mouse IgG (Cat. No. sc-2025, Santa Cruz Biotechnology) for 2 hr before addition of protein A agarose beads. After binding, beads were pelleted by centrifugation and washed with buffer. After washing, immunoprecipitated materials were eluted and immunoblotted with anti-human p300 (1:500 dilution; Cat. No. sc-584, Santa Cruz Biotechnology), anti-human CBP (1:500 dilution; Cat. No. 4772, Cell Signaling Technology), or anti-HIF-2α (1:1,000 dilution; Cat. No. NB100-132, Novus Biologicals) primary antibodies.

### *In vitro* immunoprecipitation

For the *in vitro* immunoprecipitation assays using purified proteins, we first transfected SP/HA tagged K3 or R3 human HIF-2α[8], c-myc tagged WT or HAT mouse CBP, or V5 tagged WT or MUT mouse Acss2 expression constructs into HEK293T cells; waited 48 hr; prepared whole cell extracts; incubated the ectopic HIF-2α, CBP, or Acss2 transfected cell extracts with HA (Cat. No. H9658, Sigma), c-myc (Cat. No. sc-789, Santa Cruz Biotechnology), or V5 (Cat. No. 46–0705, Invitrogen) antibodies, respectively; and then purified the ectopic proteins with protein A/G agarose (Cat. No. sc-2003, Santa Cruz Biotechnology). The ectopic proteins were eluted with 10 μg/mL working solutions of soluble competitor HA peptide (Cat. No. I2145, Sigma), c-myc peptide (Cat. No. M2435, Sigma), or V5 peptide (Cat. No. V7754, Sigma). For the *in vitro* immunoprecipitation assays, each reaction mixture contained 60 mM potassium phosphate (pH 7.5), 0.1 mM CoA, 4 mM MgCl_2_, 1 mM DTT, without or with 3 mM ATP plus 0.24 mM sodium acetate where indicated, in addition to purified CBP, Acss2 and HIF-2α ectopic proteins as specified. Where indicated, acetyl CoA was added to the final concentration instead of ATP and sodium acetate. After addition, we incubated the mixture for 30 min at 30°C, performed pulldown, and subjected the bound proteins to immunoblot analyses as indicated.

### Immunofluorescence microscopy

HT1080 cells were plated on poly-L-lysine coated coverslips and placed in sterile 24-well plates after overnight incubation under control tissue culture conditions with complete media. The next morning, plates were maintained under control, hypoxic (1% oxygen, 4 hr), low glucose (1 mM, 24 hr), or acetate (0.5 mM, 4 hr) conditions. To visualize ectopic Acss2, paraformaldehyde-fixed and Triton X-100 permeabilized cells were blocked with normal goat serum, and incubated with an anti-V5 epitope tag antibody (1:500 dilution; V5-FITC conjugate, Cat. No. R963-25, Invitrogen, Life Technologies). Nuclei were then stained with Hoechst dye (Hoechst 33258, Cat. No. 23491-45-4, Sigma). After mounting, coverslips were viewed and photographed using a fluorescent microscope (Olympus BX51, Olympus America Inc., Melville, NY). Image comparisons were made in Adobe Photoshop using identical settings for each image.

### Subcellular fractionation

To prepare subcellular fractions for knockdown/rescue HT1080 cell lines expressing WT, CYT, SV40-WT, or SV40-CYT Acss2, we used CytoBuster protein extraction reagent (Cat. No. 71009, Novagen, Gibbstown, NJ) followed by use of NE-PER nuclear and cytoplasmic extraction reagents (Cat. No. 78833, Pierce, Rockford, IL) to prepare subcellular fractions before and after the indicated treatment as previously described [10]. Samples were subjected to immunoblot analyses for fractionation. Whole cell extracts prepared in parallel were analyzed for ectopic HIF-2α acetylation as described.

### ^14^C-Acetate lipid synthesis determinations

HT1080 cells were plated as triplicates in 60 mm plates at 60% confluence and allowed to attach overnight under standard growth conditions. The next morning, cells were incubated under control (21% oxygen, 25 mM glucose), hypoxic (1% oxygen), or low glucose (1 mM glucose) conditions. Twenty-four hours later, media containing ^14^C-acetate [acetic acid, sodium salt, (1,2-^14^C); Cat. No. NEC553050UC, Perkin Elmer, Santa Clara, CA] were added and the cells grown under the same conditions for an additional 24 hr. At the time of harvest, we aspirated media, rinsed cells twice with 1x PBS, and added Triton-X 100 (0.5% in ddH_2_O) to solubilize cells. After transferring the lysis to a microfuge tube, the cellular debris was pelleted at 14,000 rpm, 4°C, 10 min, and the supernatants were transferred to a microfuge tube. We removed an aliquot for total protein determinations. For the remainder of the supernatants, we performed lipid extractions with sequential addition of methanol, chloroform, and water with vortexing and centrifugation after each step to resolve the aqueous and organic phases. After the last centrifugation, the organic phase was transferred to a new tube and evaporated to dryness under air in a hood. Extracts were resuspended in 100 μl chloroform and counted with Ecolume liquid scintillation cocktail (Cat. No. 0188247001, MP Biomedicals, Santa Ana, CA). Counts were normalized to protein concentration for each sample.

### Real-time PCR analyses

The expression of endogenous MMP9, PAI1, VEGFa, GLUT1, PGK1, and cyclophilin B in HT1080 cells were determined by reverse transcription of total RNA followed by quantitative real-time PCR analysis (qRTPCR) on an Applied Biosystems ABI Prism 7000 thermocycler using Power SYBR Green Master Mix following the manufacturer’s protocol as previously described [8]. The results of triplicate experiments, with each sample measured as triplicates, were expressed as 2^-(gene-of-interest number of cycles- cyclophilin number of cycles)^ as previously described [8]. We used the following human (forward, reverse) primer pairs: MMP9: 5′-GGGACGCAGACATCGTCATC-3′, 5′-TCGTCATCGTCGAAATGGGC-3′; PAI1: 5′-ATTCAAGCAGCTATGGGATTCAA-3′, 5′-CTGGACGAAGATCGCGTCTG-3′; VEGFa: 5′-CATCACCATGCAGATTATGCGG-3′, 5′-CCCACAGGGACGGGATTTC-3′; GLUT1: 5′-CTTTTCTGTTGGGGGCATGAT-3′, 5′-CCGCAGTACACACCGATGAT-3′; *PGK1*: 5’TTAAAGGGAAGCGGGTCGTTA-3’, 5’-TCCATTGTCCAAGCAGAATTTGA-3’; *CYCLOPHILIN B*: 5’-ATGTGGTTTTCGGCAAAGTTCTA-3’, 5’-GGCTTGTCCCGGCTGTCT-3’. We report mRNA levels relative to cyclophilin B for the indicated gene.

### Chromatin immunoprecipitation (ChIP) assays

For HT1080 cells used in ChIP experiments, we seeded 2 × 10^6^ HT1080 cells (150 mm plates) 48 hr prior to use, then exposed the cells to normoxia, hypoxia, or low glucose (1mM glucose), and finally harvested the cells for whole cell protein or RNA preparations. VEGFa, PAI1, MMP9, GLUT1, and PGK1 induction after hypoxia or low glucose exposure was confirmed by real-time RT-PCR.

Chromatin immunoprecipitation assays (ChIP) were performed using the ChIP-IT^™^ magnetic chromatin immunoprecipitation kit (Cat. No. 53008, Active Motif). The antisera for the chromatin immunoprecipitation reaction was normal mouse IgG (Cat. No. 2027, Santa Cruz Biotechnology), normal rabbit IgG (Cat. No. NI01, EMD Chemicals, Inc., Gibbstown, NJ), anti-human EPAS1 antiserum (Cat. No. NB 100–132, Novus Biologicals), anti-V5 (Cat. No. 46–0705, Invitrogen, Life Technologies), anti-human histone H3-K18ac antiserum (Cat. No. 39755, Active Motif), and anti-human histone H3-K27ac antiserum (Cat. No. 39133, Active Motif). After ChIP, precipitated genomic DNA was analyzed by quantitative PCR using an ABI Prism 7000 thermocycler (Applied Biosystems; Foster City, CA) and Power SYBR Green Master Mix (Cat. No. 4367659, Applied Biosystems) with the following human primers for the indicated regulatory regions: MMP9: 5′- GAACTTATTACGGTGCTTGACACAGT -3′ (forward) and 5′- GTATCACTCTGTCACCCAGGCTGGAGT -3′ (reverse), PAI1: 5′- GGCAGAGGGCAGAAAGGTCA -3′ (forward) and 5′- TGAACAGCCAGCGGGTCC -3′ (reverse), VEGFa: 5′- TTCCGTAGGCTAGAGTGCCC -3′ (forward) and 5′- GGTCAACACGCCAAGACATG -3′ (reverse), GLUT1: 5′- GGGCTGTCTTACTCACTCTTACTCC -3′ (forward) and 5′- CTCTTCTGGGTTGTGTTCAAGCTG -3′ (reverse), PGK1: 5′- GGATCTTCGCCGCTACCCTTGTG -3′ (forward) and 5′- CTATTGGCCACAGCCCATCGCGGTC -3′ (reverse), RPL13A: 5′- GAGGCGAGGGTGATAGAG -3′ (forward) and 5′- ACACACAAGGGTCCAATTC -3′ (reverse). Captured genomic DNA was normalized to input material, and then the normoxic, hypoxic, and low glucose samples were compared.

Sequential chromatin immunoprecipitation assays were performed using the Re-ChIP-IT^™^ magnetic chromatin re-immunoprecipitation kit (Cat. No. 53016, Active Motif). The antisera for the first chromatin immunoprecipitation reaction was normal mouse IgG (Cat. No. 2027, Santa Cruz Biotechnology) or anti-human EPAS1 antiserum (Cat. No. NB 100–132, Novus Biologicals). The antisera for the second chromatin immunoprecipitation reaction was anti-human CBP (Cat. No. 7389, Cell Signaling Technology). After sequential ChIP, the precipitated genomic DNA was analyzed by quantitative PCR as described above.

### Histone H3 analyses

HT1080 cells were harvested, washed twice with ice-cold PBS supplemented with 5 mM sodium butyrate, and counted. Then 10^7^ cells were resuspended and lysed in 1 mL Triton extraction buffer [TEB: PBS containing 0.5% Triton X 100 (v/v), 2 mM phenylmethylsulfonyl fluoride (PMSF), 0.02% (w/v) NaN_3_] on ice for 10 minutes with mixing by gentle inversion. Cell lysates were centrifuged at 2000 rpm for 10 min at 4°C, and pellets were washed once by centrifugation in 500 uL TEB. The nuclear pellets were resuspended in 250 uL 0.2 N HCl and rotated overnight at 4°C. The supernatant containing histones was removed after centrifugation and 1/5 volume NaOH was added to neutralize the pH. The protein concentration was determined by Bradford assay.

Histones were evaluated by immunoblot analysis using antibodies for the following antigens: pan histone H3 (1:500 dilution; Cat. No. 61278, Active Motif), histone H3-K9ac (1:500 dilution; Cat. No. 39138, Active Motif), histone H3-K14ac (1:500 dilution; Cat. No. 39670, Active Motif), histone H3-K18ac (1:500 dilution; Cat. No. 39756, Active Motif), histone H3-K27ac (1:500 dilution; Cat. No. 39136, Active Motif), histone H3-K9me3 (1:500 dilution; Cat. No. 39162, Active Motif), histone H3-K27me3 (1:500 dilution; Cat. No. 39157, Active Motif), or histone H3-R17me2 (1:500 dilution; Cat. No. 39710, Active Motif).

### Cell proliferation assays

For cell proliferation assays, 1 x 10^3^ HT1080 cells/well were seeded in a 96-well plate with each cell line in 8-well replicate sets. After 24 h, cells were exposed for 1 week to 1% oxygen with complete (25 mM glucose) media, or exposed to 21% oxygen with either complete media, low glucose (1 mM glucose) media, or complete media supplemented with acetate (5 mM). Media were changed every 48 hr with comparable media. Cell proliferation was detected every day with the CellTiter 96 AQueous Non-Radioactive Cell Proliferation Kit (Cat. No. G5421, Promega, Madison, WI).

### Colony formation assays

For colony formation assays, 5 x 10^2^ HT1080 cells seeded in triplicate 100 mm plates were allowed to attach for 24 hr in complete medium. After 24 hr, medium was changed and cells were cultured for 10 days under control (21% O_2_, complete medium), hypoxia (1% O_2_, complete medium), low glucose (21% O_2_, low glucose medium) or acetate supplemented (21% O_2_, complete medium with 5 mM acetate) conditions. Media was not changed throughout the experiment. Colonies were stained with 1% crystal violet in ethanol/PBS (15%/85%). Cells were imaged and colony number determined using ImageJ software.

### Cell migration and cell invasion assays

For cell migration assays, HT1080 cells were serum-starved in 0.5% FBS/DMEM media overnight. After 12 hr, 1.5 x 10^5^ HT1080 cells in serum-free media were transferred into a transwell insert. For cells maintained under normal conditions, cells were incubated with complete media and exposed to 21% oxygen for 4 h. For cells maintained under hypoxic conditions, cells were incubated with complete media and exposed to 1% oxygen for 4 hr. For cells exposed to low glucose conditions, cell were incubated with low glucose (1 mM) media at 21% oxygen for 24 hr and compared to control cells maintained under standard glucose (25 mM) conditions for 24 hr. For acetate-supplementation assays, cells were incubated with complete media supplemented with acetate (5 mM) and exposed to 21% oxygen for 4 h. Cell migration was detected in triplicates for each treatment after crystal violet staining. The absorbance was recorded at 560 nm with a microplate reader.

For cell invasion assays, we used a commercially available kit containing wells pre-filled with Matrigel (CytoSelect 24-Well Cell Invasion Assay Kit; Cat. No. CBA-110, Cell Biolabs, San Diego, CA). HT1080 cells were serum-starved in 0.5% FBS/DMEM media overnight. After 12 hr, 1.5 x 10^5^ HT1080 cells in serum-free media were transferred into the transwell insert as above after pre-incubating the transwell insert for 1 hr with serum-free media at room temperature. Cell migration was determined from triplicates for each treatment according to the manufacturer’s protocol.

### *In vivo* nude mice flank tumor experiments

All animal experiments were approved by the UTSWMC Institutional Animal Care and Use Committee. Mice were anesthetized by isoflurane inhalation prior to flank tumor injections. Female nude mice obtained from NCI were injected subcutaneously on the left dorsal flank with 5×10^6^ luciferase-expressing stably transformed HT1080 cells suspended in 0.5 ml DMEM, that were grown using 3% FBS to increase subsequent *in vivo* tumor cell growth and seeding efficiency. Tumor sizes for volume estimates were measured using calipers every other day beginning on the fourth day after cell injections. Beginning six days after injection, mice were administered vehicle (PBS, 0.01 mL/g body weight) or glyceryl triacetate (GTA; 90 μL/25 gm body weight; Cat. No. W200700, Sigma-Aldrich Chemicals, Saint Louis, MO) by oral gavage once per day [15]. All mice were harvested at the same day following tumor implantation when tumor volumes reached ~1.5 cm^3^ for at least 25% of the mice in the largest tumor-bearing set. Mice were monitored on a daily basis following tumor implantation for signs of distress. Any mice meeting euthanasia or tumor size criteria were anesthetized by isoflurane inhalation and euthanized by bilateral thoracotomy prior to lung as well as tumor excision.

### *Ex vivo* nude mice flank tumor experiments

Mice were sacrificed, and lungs as well as primary tumors removed for biochemical luciferase activity determination. Individual tissues were weighed and homogenized using a PowerGen 700D homogenizer (ThermoFisher Scientific, Waltham, MA) in lysis reagent (25 mM Tris-phosphate pH 7.8, 2 mM DTT, 2mM 1,2 diaminocyclohexane-N,N,N,N-tetra-acetic acid, 10% glycerol, 1% NP-40) containing soybean trypsin inhibitor (0.2 mg/ml) and bovine serum albumin (0.2 mg/ml). Duplicate samples (2 μl tumor or 20 μl lung lysates) were diluted in 100 μl lysis reagent containing 2.5 mM MgCl_2_. Immediately prior to measurement, 50 μl luciferin reagent (20 mM tricine, 1 mM (MgCO_3_)_4_Mg(OH)_2_.5H_2_O, 2.67 mM MgSO_4_, 0.1 mM EDTA, 33 mM DTT, 0.27 mM coenzyme Q, 0.47 mM luciferin, 0.53 mM ATP, pH 7.8) was added and measurement performed for 10 sec in a single-tube luminometer (Sirius, Berthold Detection Systems, Pforzheim, Germany).

### Statistical analyses

We compared results obtained from the indicated experimental groups by unpaired Student’s t-Test with Welch’s correction for groups of equal sample size or by z-Test for groups of unequal sample size. One-tailed or two-tailed analyses were performed as indicated. We assumed equal variances for experimental groups. We used one-way or two-way ANOVA analyses for multiple comparisons with Dunnett’s multiple comparison posthoc test. The statistical analyses were performed using StatPlus (AnalystSoft Inc.) and Prism 7 (GraphPad Software, Inc.). All P values less than or equal to 0.05 (*) or 0.10 (**) are reported for the stated comparisons.

## Results

### Specific HIF-2α lysine residues are required for Cbp/HIF-2α interactions

**Fig 1:**
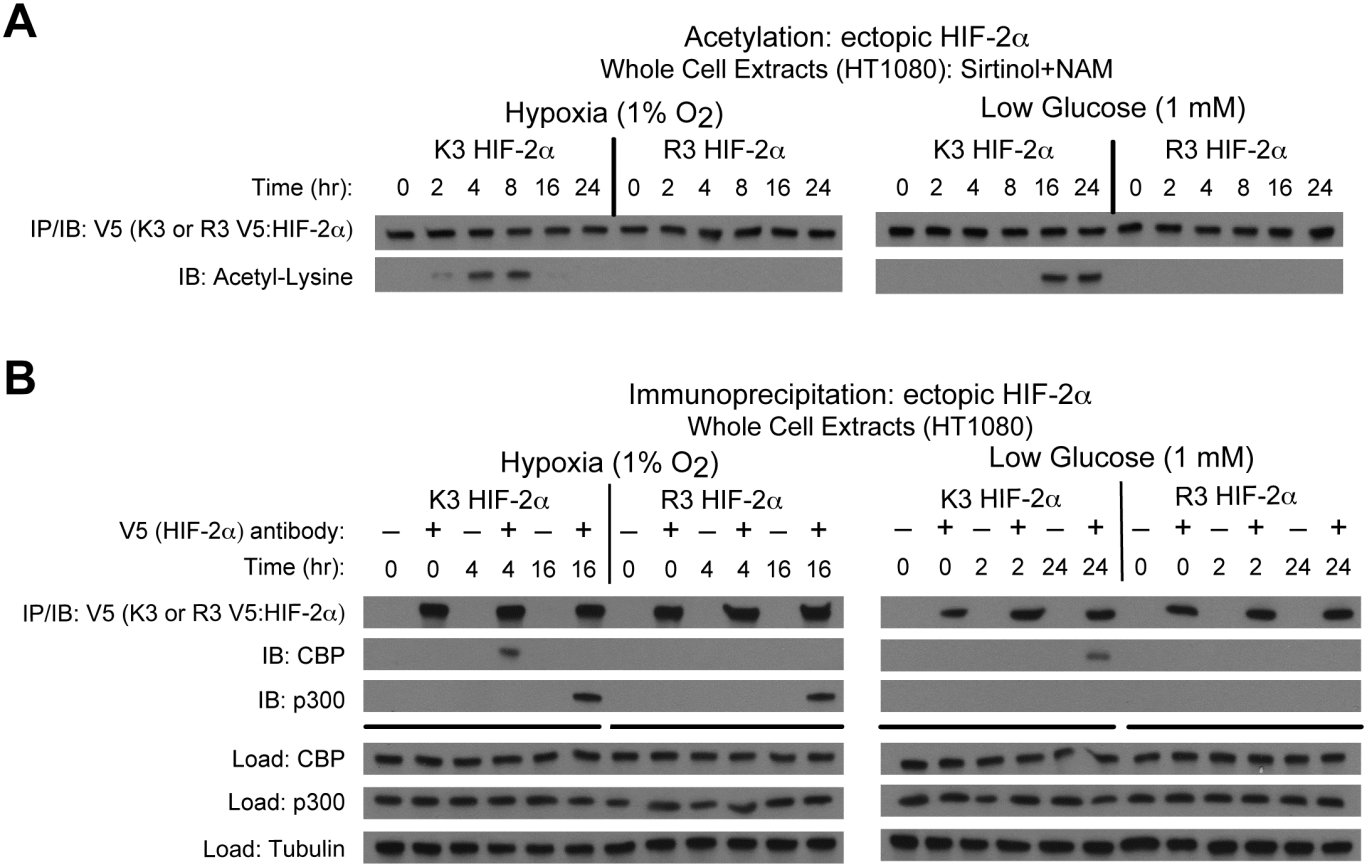
Specific HIF-2α lysine residues are required for Cbp/HIF-2α interactions. (A) Acetylation of ectopic HA-tagged wild-type (K3) or arginine-lysine substituted mutant (R3) HIF-2α detected by immunoblotting (IB) with anti-V5 or anti-acetylated lysine antibodies following immunoprecipitation (IP) after hypoxia or low glucose exposure. (B) Interactions of endogenous Cbp or p300 with ectopic K3 or R3 HIF-2α after (0, 4, 16 hr) hypoxia or (0, 2, 24 hr) low glucose exposure.

Endogenous HIF-2α forms stable complexes with Cbp in an Acss2-dependent manner [14, 16]. We asked whether specific HIF-2α lysine residues, which are acetylated by Cbp during hypoxia [9], are required for Cbp/HIF-2α complex formation induced by hypoxia or glucose deprivation. We generated stably transformed knockdown HT1080 cells retaining or lacking endogenous HIF-2α, and expressing ectopic HIF-2α with intact lysine residues acetylated by Cbp (K3) or a HIF-2α arginine substitution mutant that is not acetylated by and unable to interact with Cbp (R3) [8, 9]. Under stress conditions, K3, but not R3, HIF-2α is acetylated in a temporal manner (Fig 1A). K3, but not R3, HIF-2α complexes with Cbp at identical time-points observed with endogenous HIF-2α, whereas complex formation with p300 is unaffected by mutations in these specific HIF-2α lysine residues (Fig 1B)[14].

### Acss2-generated acetyl CoA controls Cbp/HIF-2α interactions

Cbp/HIF-2α complex formation can be induced *in vitro* by addition of Acss2 substrate and cofactor, acetate and ATP, or by addition of Acss2 product, acetyl CoA, when wild-type Acss2 is present [16]. Cbp/HIF-2α acetylation requires HIF-2α lysines residues, Cbp acetyltransferase activity, and the acetyl CoA generator Acss2 [8, 9, 14, 16]. We asked whether alterations in any of these factors would affect Cbp/HIF-2α complex formation in this assay.

**Fig 2:**
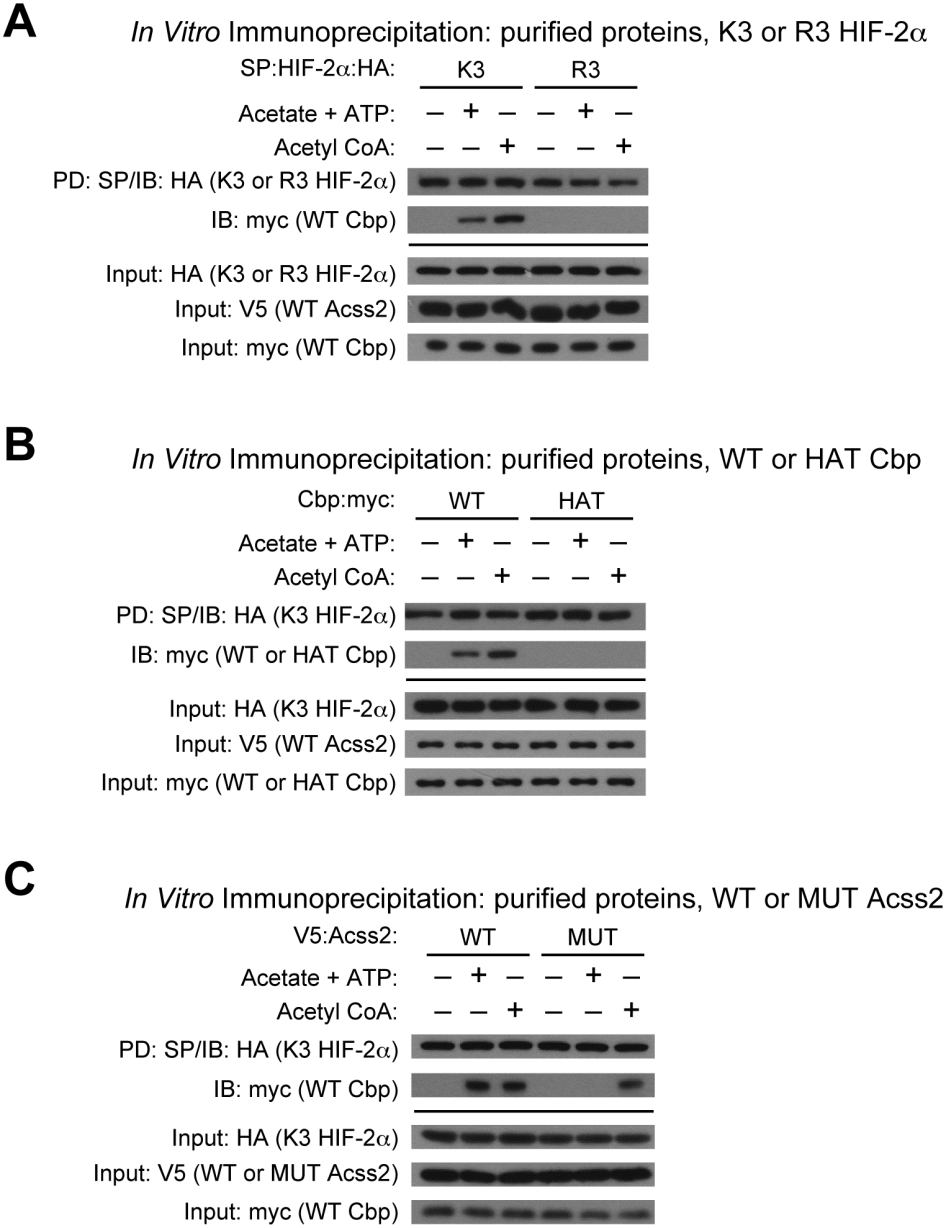
Acss2-generated acetyl CoA controls Cbp/HIF-2α interactions. (A) Interaction of ectopic SP- and HA-tagged wild-type (K3) or arginine-lysine substituted mutant (R3) HIF-2α with myc-tagged wild-type (WT) Cbp detected by immunoblotting (IB) with anti-HA or anti-myc antibody following pulldown (PD) with SP-agarose after incubation with acetate +ATP or with acetyl CoA in the presence of purified V5-tagged wild-type (WT) Acss2. (B) Interaction of ectopic SP- and HA-tagged K3 HIF-2α with myc-tagged WT or histone acetyltransferase mutant (HAT) Cbp detected by immunoblotting with anti-HA or anti-myc antibody following pulldown with SP-agarose after incubation with acetate +ATP or with acetyl CoA in the presence of purified V5-tagged WT Acss2. (C) Interaction of ectopic SP- and HA-tagged K3 HIF-2α with myc-tagged WT Cbp detected by immunoblotting with anti-HA or anti-myc antibody following pulldown with SP-agarose after incubation with acetate +ATP or with acetyl CoA in the presence of purified V5-tagged WT or mutant (MUT) Acss2.

Consistent with HIF-2α acetylation and complex formation Cbp being linked processes, K3, but not R3, HIF-2α complexes with Cbp upon addition of acetate/ATP or acetyl CoA (Fig 2A). Similarly, enzymatically active Cbp is required for stable Cbp/HIF-2α complex formation as wild-type (WT), but not histone acetyltransferase mutant (HAT), Cbp complexes with K3 HIF-2α upon addition of acetate/ATP or acetyl CoA (Fig 2B). Finally, stable Cbp/HIF-2α complexes form in the presence of wild-type (WT), but not enzymatically inactive mutant (MUT), Acss2 when supplied with acetate/ATP, whereas the addition of the Acss2 end product acetyl CoA results in stable Cbp/HIF-2α complex formation in the presence of either WT or MUT Acss2 (Fig 2C).

### A mutation in a putative nuclear localization signal impairs Acss2 translocation

**Fig 3:**
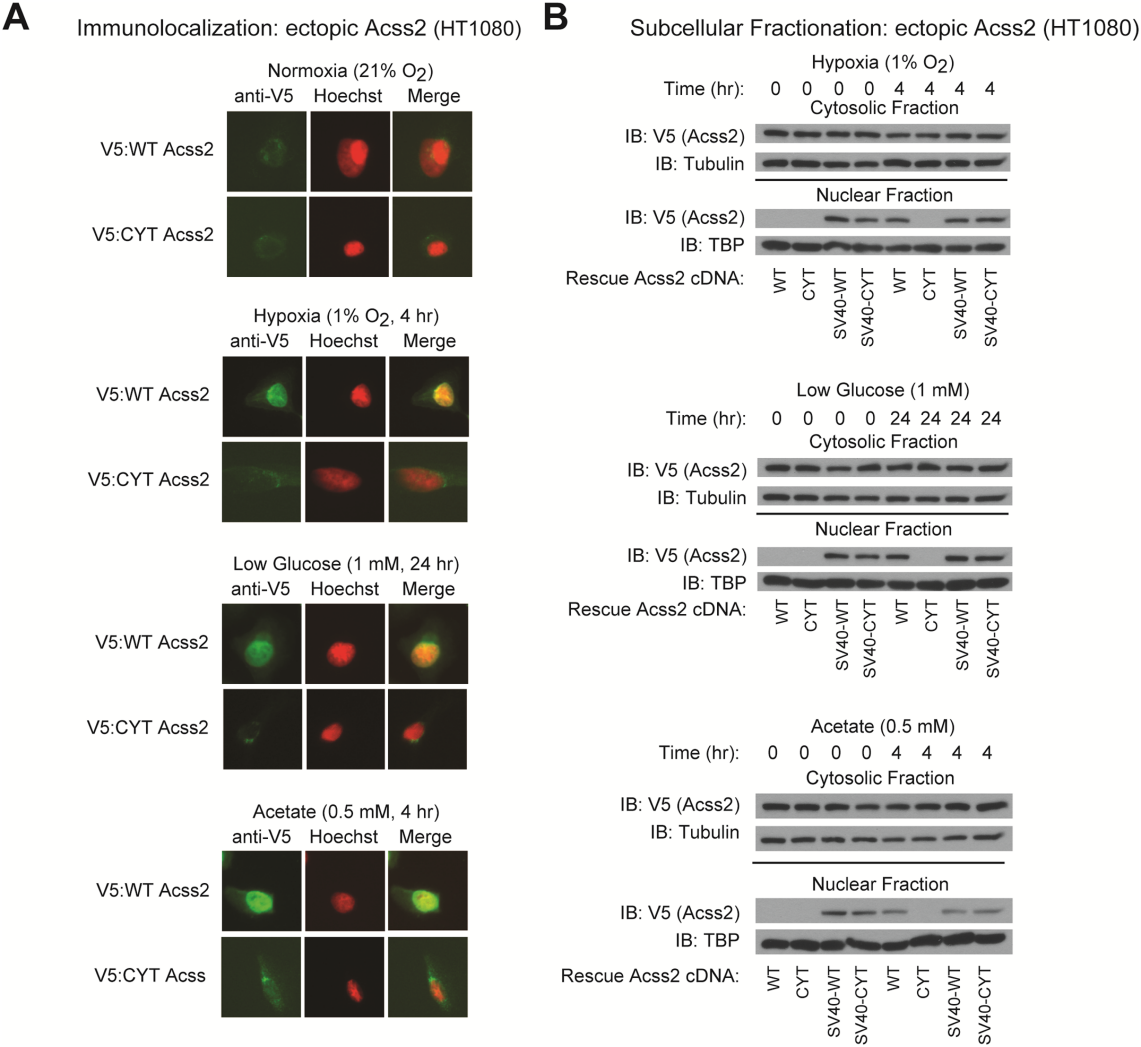
A mutation in a putative nuclear localization signal impairs Acss2 translocation. (A) Subcellular localization of ectopic V5-tagged wild-type (WT) or cytosol-restricted mutant (CYT) Acss2 in HT1080 cells under basal and stress conditions as detected by immunofluorescence and merging with Hoechst-stained cells to detect nuclei. (B) Subcellular fractionation of stably transformed HT1080 cells with knockdown of endogenous Acss2 and rescue with ectopic WT or CYT mutant Acss2 without or with an SV40 nuclear localization signal fused to the amino terminus. Studies were performed under hypoxia, low glucose, or acetate exposure for the indicated periods.

We hypothesized that Acss2, which transits to the nucleus under stress conditions [14, 16], produces acetyl CoA used for nuclear HIF-2α acetylation and Cbp/HIF-2α complex formation. We identified a putative nuclear localization signal (NLS) in a region of wild-type (WT) mouse Acss2 containing basic residues. We generated a substitution mutant (CYT) using residues present in this region of a prokaryotic Acss2 homologue. CYT Acss2 does not translocate to the nucleus following exposure to hypoxia, glucose deprivation, or exogenous acetate as assessed by immunofluorescence (Fig 3A and S1 Fig). WT and CYT Acss2 are not detected in the nucleus of cells maintained under basal conditions (Fig 3A).

We also used cell fractionation to determine if CYT Acss2 can transit to the nucleus. Similar to the immunofluorescence results, CYT Acss2 is not found in the nucleus following stress induced by hypoxia or glucose deprivation as well as following addition of exogenous acetate (Fig 3B). Forced nuclear targeting of WT or CYT Acss2 under basal as well as stress conditions is conferred by fusion of the SV40 large T antigen nuclear localization signal (SV40-NLS) to the Acss2 amino terminus (Fig 3B).

### Cytosol-restricted Acss2 is enzymatically active

**Fig 4:**
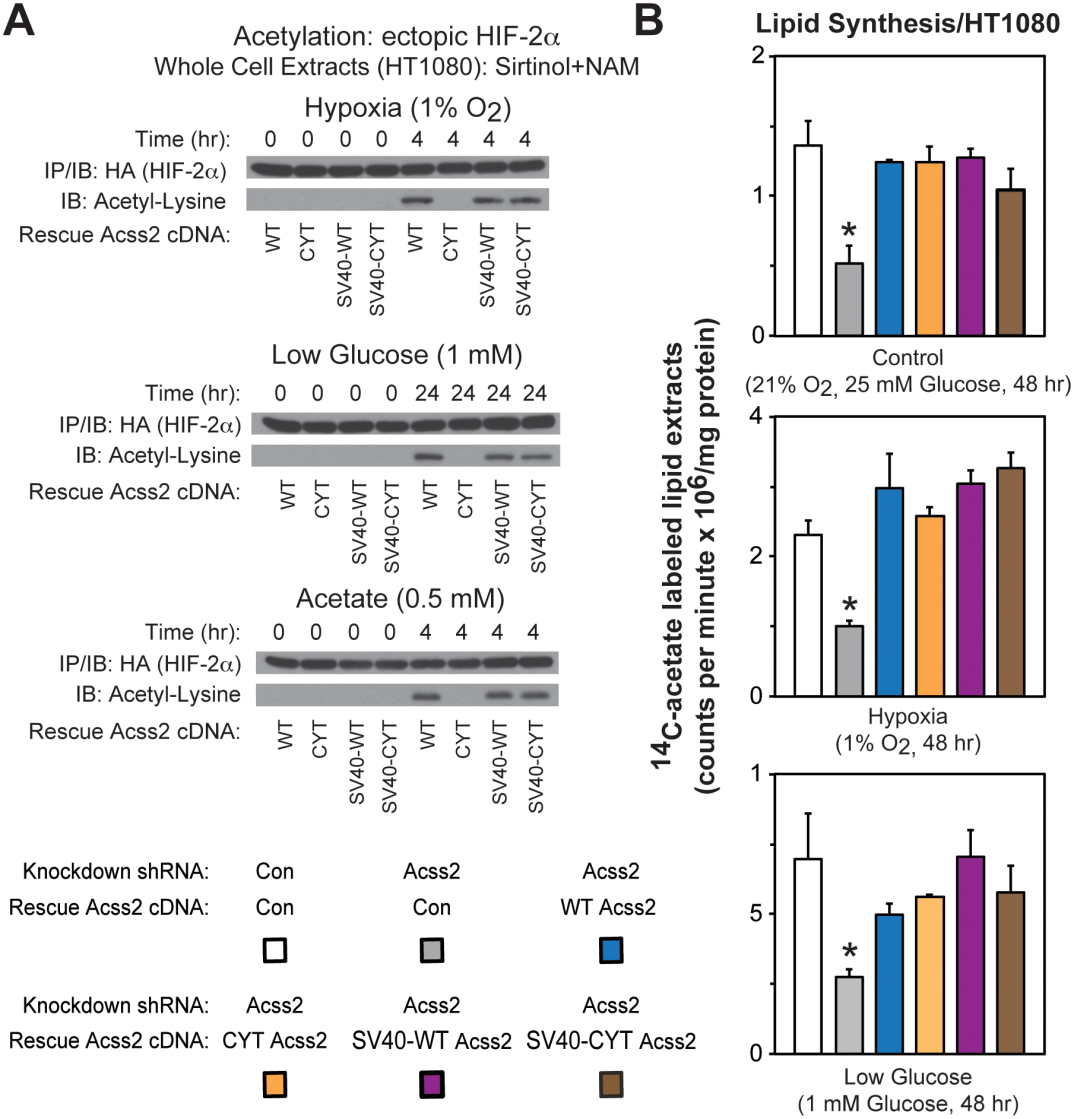
Cytosol-restricted Acss2 is enzymatically active. (A) Acetylation of ectopic HA-tagged HIF-2α detected by immunoblotting (IB) with anti-HA or anti- acetylated lysine antibodies following immunoprecipitation (IP) with anti-HA antibody in stably transformed HT1080 cells with knockdown of endogenous Acss2 and rescue with ectopic wild-type (WT) or cytosol-restricted mutant (CYT) Acss2 without or with an SV40 nuclear localization signal fused to the amino terminus. Studies were performed under hypoxia, low glucose, or acetate exposure for the indicated periods. (B) Acetate-dependent lipid synthesis measured by ^14^C-acetate incorporation in HT1080 stably-transformed cells producing control or Acss2 shRNA downstream of a luciferase cDNA cassette and expressing ectopic control, WT, CYT, SV40-WT, or SV40-CYT Acss2. Cells were incubated under (A) control, (B) hypoxic, or (C) low glucose conditions for 48 hr with labeling performed during the last 24 hr. Comparison of samples within a given condition was made by one-way ANOVA followed by Dunnett’s multiple comparisons test using control shRNA knockdown/control rescue as reference with decreased samples noted (*, P<0.05). All values are means with SD.

We hypothesize that HIF-2α acetylation requires enzymatically active Acss2 be present in the nucleus when acetate levels are increased. We therefore asked whether CYT Acss2 facilitates HIF-2α acetylation. WT, but not CYT, Acss2 confers HIF-2α acetylation following exposure to hypoxia, glucose deprivation, or exogenous acetate (Fig 4A). Despite constitutive localization, HIF-2α acetylation by SV40 NLS-Acss2 fusion proteins is only evident under stress conditions or upon acetate addition (Fig 4A). The CYT mutation likely has selective effects on acetylation since nearly complete depletion of Acss2 does not affect global acetylation under basal, hypoxia, or glucose deprivation conditions (S2 Fig).

CYT Acss2 retains acetyl CoA generating capacity when force-translocated into the nucleus. To assess whether CYT Acss2 retains acetyl CoA generating capacity in the cytosol, we also measured acetate-dependent lipid synthesis rates under control, hypoxia, or glucose deprivation conditions. Consistent with CYT Acss2 being only defective in nuclear localization and not in acetyl CoA generating capacity, all four rescue Acss2 forms (WT, CYT, SV40-WT, SV40-CYT) retain acetate-dependent cytosolic lipid synthesis capacity, which is otherwise blunted by Acss2 knockdown (Fig 4B).

### Maximal HIF-2 signaling during stress requires HIF-2α acetylation

**Fig 5:**
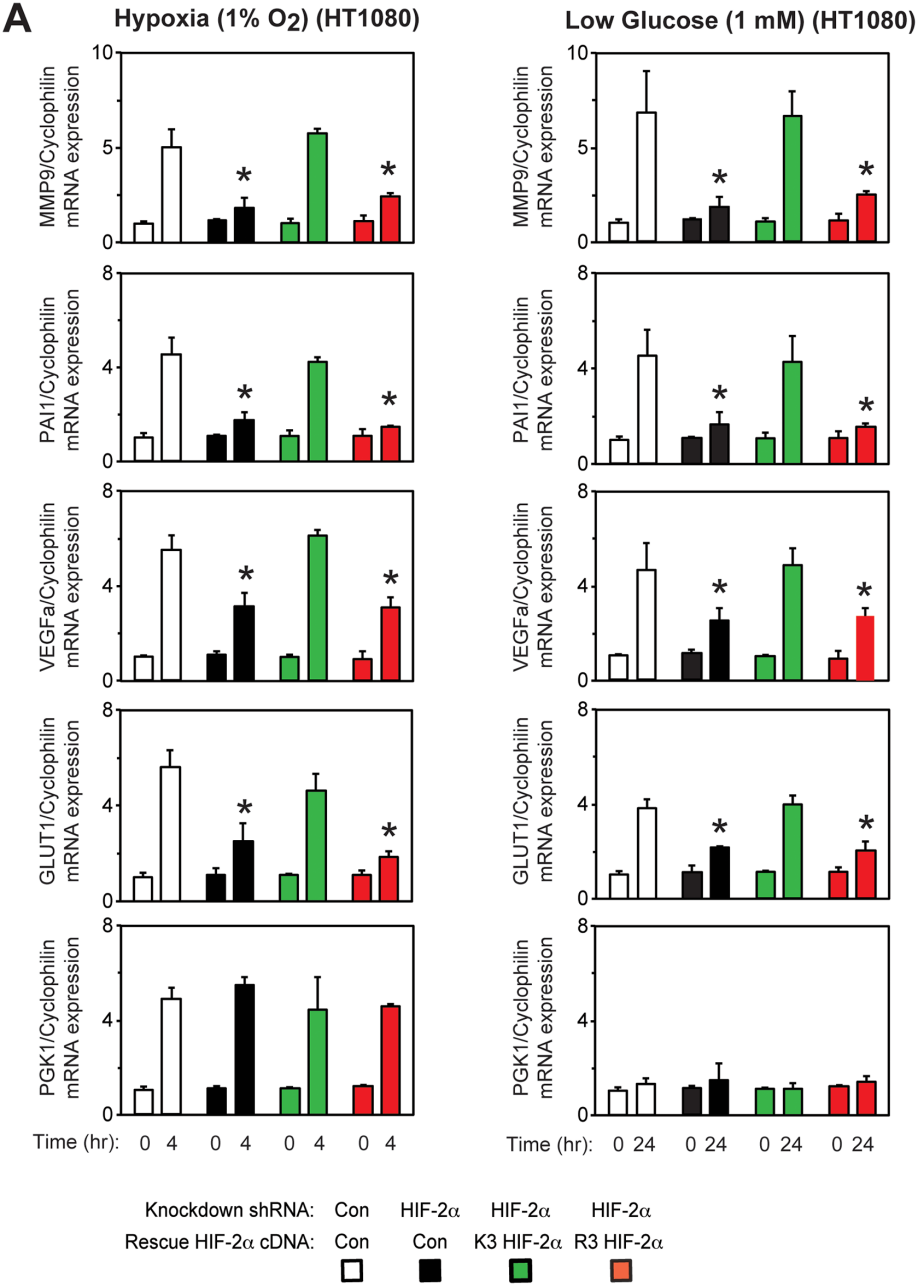
Maximal HIF-2 signaling during stress requires HIF-2α acetylation. Semi-quantitative RTPCR measurement of HIF-1 selective (PGK1), HIF-2 preferential (MMP9, PAI1), or HIF-1/HIF-2 co-regulated (VEGFa, GLUT1) target genes in stably transformed control or HIF-2α shRNA knockdown HT1080 cells, or stably transformed knockdown/rescue HT1080 cells expressing ectopic wild-type (K3) or arginine-lysine substituted mutant (R3) HIF-2α after (A) early (4 hr) hypoxia or (B) late (24 hr) low glucose treatment. Comparison of samples within a given condition was performed by one-way ANOVA followed by Dunnett’s multiple comparisons test using control shRNA knockdown/control rescue as reference with decreased samples noted (*, P<0.05). Values indicated are means with SD.

Specific HIF-2α lysine residues, which are acetylated by Cbp during hypoxia[9], are required for stable Cbp/HIF-2α complex formation induced by hypoxia or glucose deprivation (Fig 1). We measured HIF target gene induction in stably transformed knockdown/rescue HT1080 cells expressing ectopic K3 or R3 HIF-2α under control or stress conditions (hypoxia or glucose deprivation). K3 HIF-2α efficiently induces HIF-2 target genes associated with tumor growth and metastasis (MMP9, PAI1, VEGFa, GLUT1) during hypoxia (Fig 5A) and low glucose (Fig 5B) stress conditions. In comparison, HIF-2 target gene induction is blunted in R3 HIF-2α knockdown/rescue and HIF-2α knockdown cells. Induction of the HIF-1 target gene PGK1 is unaffected in K3 or R3 HIF-2α knockdown/rescue cells as well as in HIF-2α knockdown cells.

### Maximal HIF-2 signaling during stress requires nuclear Acss2

**Fig 6:**
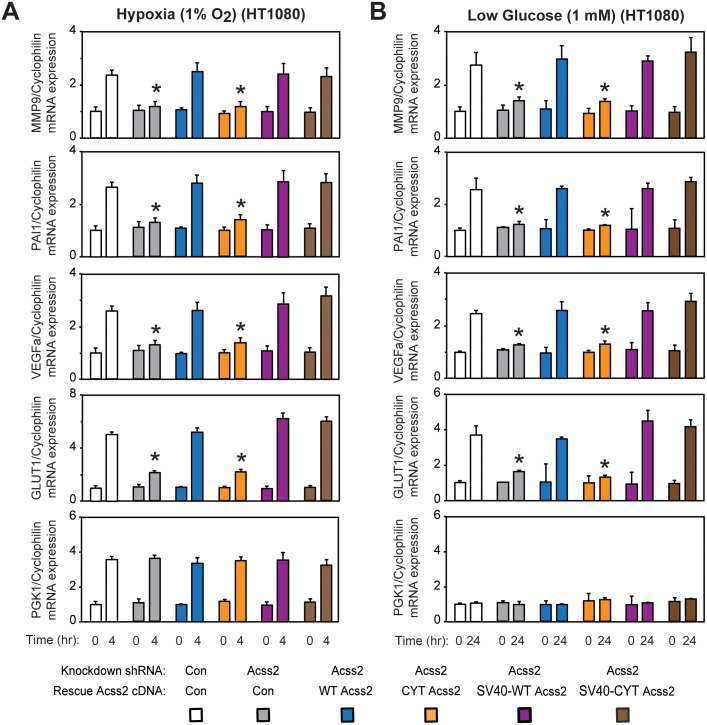
Maximal HIF-2 signaling during stress requires nuclear Acss2. Semi-quantitative RTPCR measurement of HIF-1 selective (PGK1), HIF-2 preferential (MMP9, PAI1), or HIF-1/HIF-2 co-regulated (VEGFa, GLUT1) target genes in stably transformed control or Acss2 shRNA knockdown HT1080 cells, or stably transformed knockdown/rescue HT1080 cells expressing ectopic wild-type (WT) or cytosol-restricted mutant (CYT) Acss2 without or with an SV40 nuclear localization signal fused to the amino terminus after (A) early (4 hr) hypoxia or (B) late (24 hr) low glucose treatment. Comparison of samples within a given condition was performed by one-way ANOVA followed by Dunnett’s multiple comparisons test using control shRNA knockdown/control rescue as reference with decreased samples noted (*, P<0.05). Values indicated are means with SD.

We reasoned that HIF-2 stress signaling requires Acss2 be present in the nucleus when acetate levels are increased. We measured HIF target gene induction in stably transformed knockdown/rescue HT1080 cells expressing ectopic WT, CYT, SV40-WT, or SV40-CYT Acss2 under control or stress conditions (hypoxia or glucose deprivation). Only CYT Acss2 knockdown/rescue cells are impaired in their ability to induce HIF-2 target genes under hypoxia (Fig 6A) or glucose deprivation (Fig 6B) conditions, which are also blunted in Acss2 knockdown cells. Induction of HIF-2 target genes in WT, SV40-WT, or SV40-CYT Acss2 knockdown/rescue cells is evident only under stress conditions, consistent with signaling induced coincident with increased acetate levels.

### Acetylated HIF-2α regulates Cbp-mediated stress remodeling

**Fig 7:**
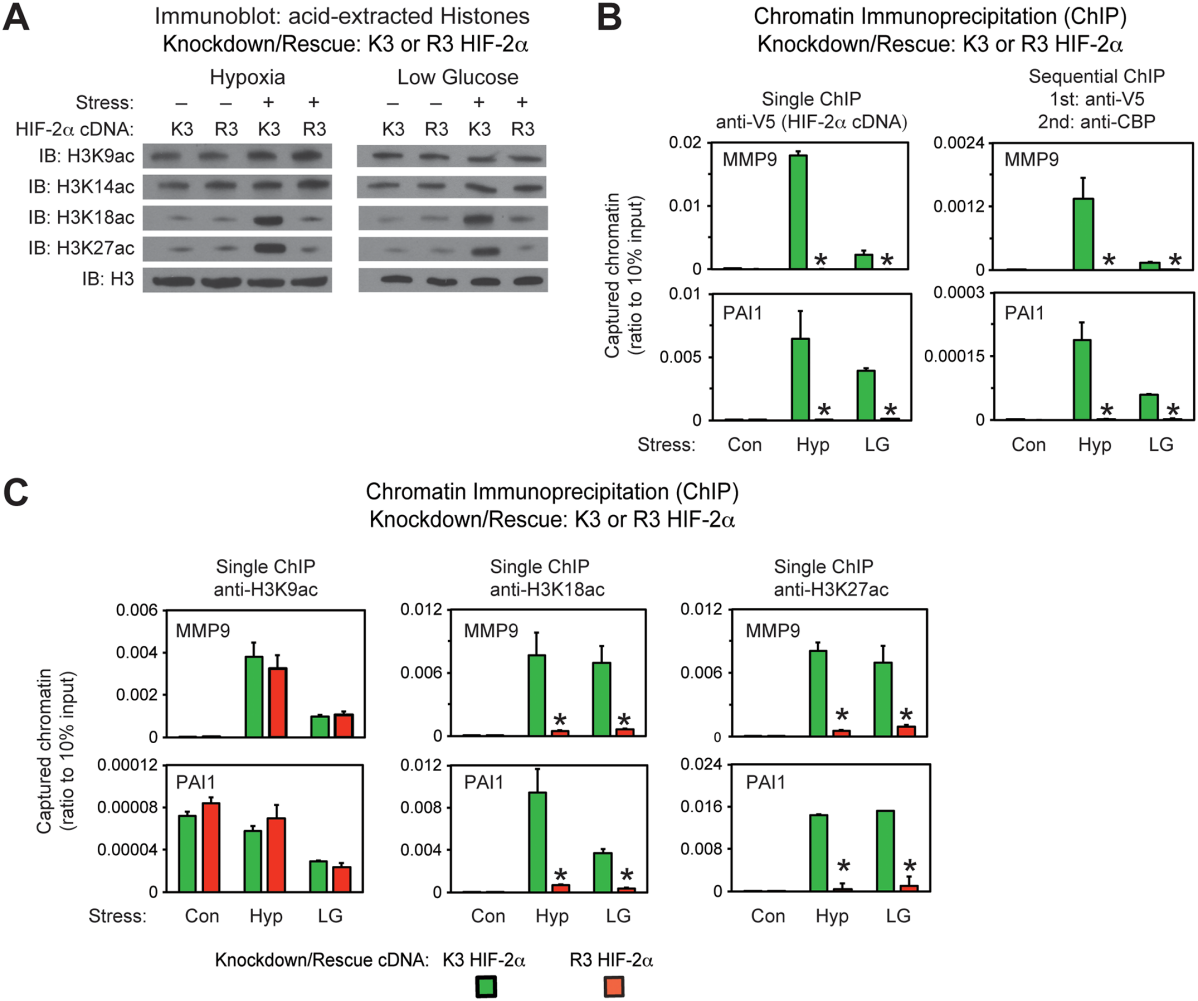
Acetylated HIF-2α regulates Cbp-mediated stress remodeling. (A) Assessment of global H3K9ac, H3K14ac, H3K18ac and H3K27ac acetylation epigenetic marks in stably transformed K3 or R3 HIF-2α knockdown/rescue HT1080 cells with knockdown of endogenous HIF-2α and rescue with ectopic V5-tagged wild-type (K3) or arginine-lysine substituted mutant (R3) HIF-2α maintained under control (Con) conditions or following exposure to early (4 hr) hypoxia (Hyp) or late (24 hr) low glucose (LG) conditions. (B) Single and sequential chromatin immunoprecipitation (ChIP) assays in stably transformed K3 or R3 HIF-2α knockdown/rescue HT1080 cells used in (A) under control (Con), hypoxia (Hyp), or low glucose (LG) conditions. The single and first stage of the sequential ChIP was performed with antibodies recognizing V5. The second stage of the sequential ChIP was performed with antibodies recognizing endogenous Cbp. The amplicons detect chromatin containing HIF-responsive elements (HRE) in regulatory regions of the HIF-2 target genes MMP9 and PAI1. (C) Single ChIP assays in same cells and with same amplicons as in (B), but using antibodies recognizing specific acetylation marks in histone 3 induced by Cbp, H3K18ac and H3K27ac, as well as a histone 3 mark not modified by Cbp, H3K9ac. Comparison of samples within a given condition was performed by one-tailed unpaired t test with decreased samples noted (*, P<0.05). Values indicated are means with SD.

How epigenetic modifications are altered during stress is largely unknown, including for histone acetylation [17–19]. Nearly half of all HIF-1 target genes require Cbp and p300 for activation [20]. During hypoxia, HIF-1 and HIF-2 physically associate with a large numbers of loci, but HIF-2 plays a transcriptional role in only a minor portion, suggesting an alternative function for HIF-2 [21, 22]. We asked if altering Cbp/HIF-2α interactions as a result of mutations in HIF-2α acetylated residues affects histone 3 epigenetic marks. Global H3K18ac and H3K27ac levels increase in K3, but are unchanged in R3, HIF-2α knockdown/rescue cells (Fig 7A). Epigenetic marks associated with other modifying enzymes (H3K9ac, H3K14ac, H3R17me2), poised enhancers (H3K9me3, H3K27me3), or histone 3 (pan histone3) levels are grossly unchanged in K3 or R3 HIF-2α knockdown/rescue cells (Fig 7A and S3 Fig).

We next asked whether determinants of Cbp/HIF-2α complex formation in solution also apply at the chromatin level. Efficient recruitment of acetylation-intact (K3), but not of acetylation-defective (R3), HIF-2α to promoter regions of HIF-2 target genes (MMP9, PAI1, VEGFa, GLUT1) is observed after either hypoxia or low glucose exposure as assessed by single chromatin immunoprecipitation (ChIP); in addition, sequential ChIP reveals that Cbp is also recruited with K3 HIF-2α to HIF-2 regulatory elements (Fig 7B and S4A Fig). Interestingly, Cbp is not detected by sequential ChIP at HIF-1 (PGK1) dependent regulatory elements, even when single ChIP detects HIF-2 (S4A Fig).

Cbp and HIF-2α act in concert to form complexes on chromatin. Cbp also acetylates histone 3 proteins at specific marks, H3K18ac [23, 24] and H3K27ac [25]. We asked if disrupting Cbp/HIF-2α interactions, through mutations of specific HIF-2α lysine residues required for stable Cbp/HIF-2α complex formation, also perturbs these histone marks at promoter regions of specific HIF-2 target genes (MMP9, PAI1, VEGFa, GLUT1). H3K18ac and H3K27ac epigenetic marks at these HIF-2 regulatory regions increase during stress in K3 HIF-2α knockdown/rescue cells, but are reduced in R3 HIF-2α knockdown/rescue cells (Fig 7C and S4B Fig). These epigenetic marks are not decreased in a HIF-1 regulatory region (PGK1) or a non-HIF promoter (RPL13A) for R3 HIF-2α knockdown/rescue cells (S4B Fig). Acetylation of a histone 3 residue (H3K9ac) modulated by other modifying enzymes besides Cbp is not decreased in R3 HIF-2α knockdown/rescue cells at HIF-2, HIF-1, or non-HIF associated chromatin regions (S4B Fig).

### Nuclear Acss2 regulates Cbp-mediated stress remodeling

**Fig 8:**
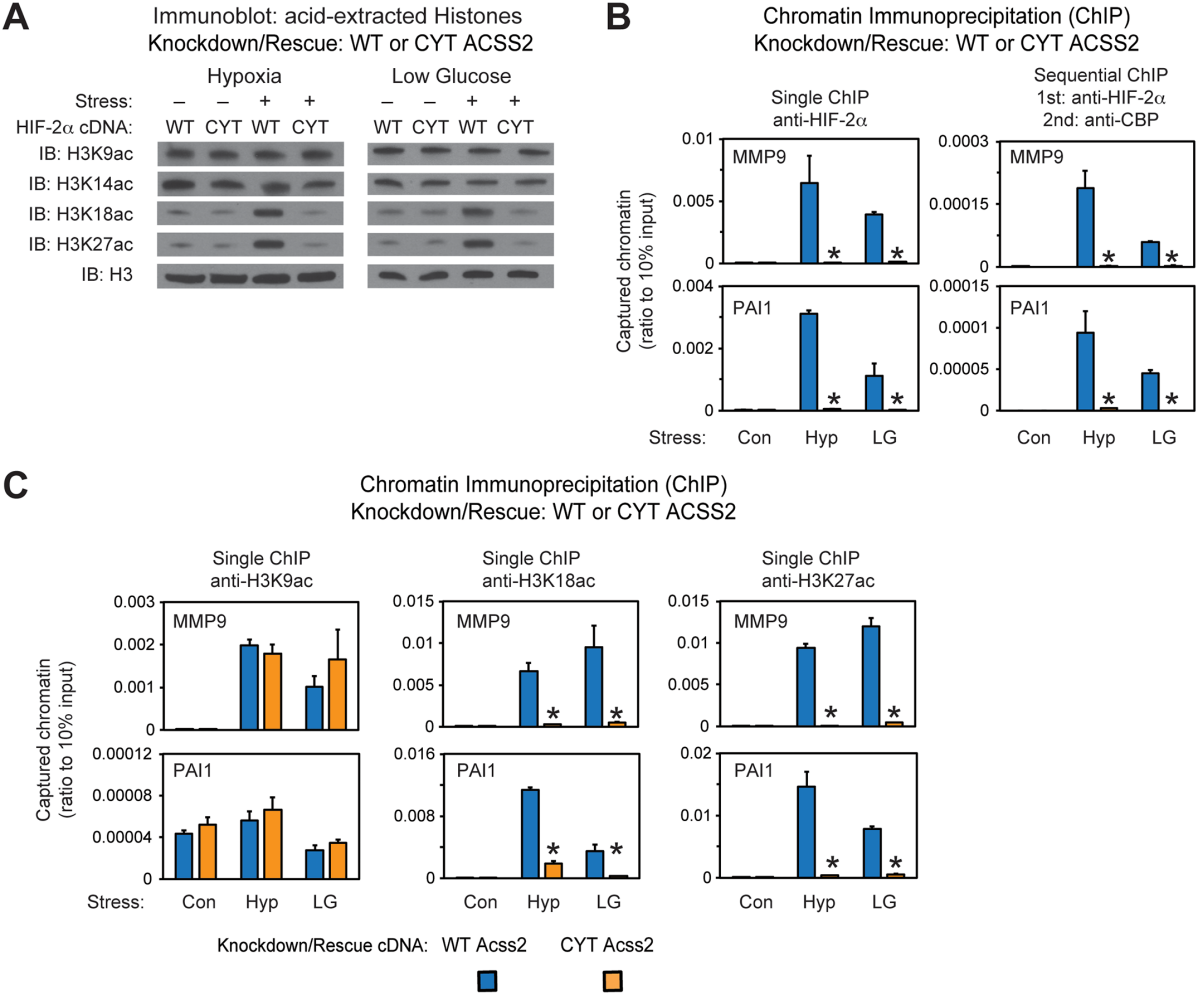
Nuclear Acss2 regulates Cbp-mediated stress remodeling. (A) Assessment of global H3K18ac and H3K27ac acetylation epigenetic marks in stably transformed WT or CYT Acss2 knockdown/rescue HT1080 cells with knockdown of endogenous Acss2 and rescue with ectopic wild-type (WT) or cytosol-restricted mutant (CYT) Acss2 maintained under control (Con) conditions or following exposure to early (4 hr) hypoxia (Hyp) or late (24 hr) low glucose (LG) conditions. (B) Single and sequential chromatin immunoprecipitation (ChIP) assays in stably transformed WT or CYT Acss2 knockdown/rescue HT1080 cells used in (A) under control (Con), hypoxia (Hyp), or low glucose (LG) conditions. The single and first stage of the sequential ChIP was performed with antibodies recognizing endogenous HIF-2α. The second stage of the sequential ChIP was performed with antibodies recognizing endogenous Cbp. The amplicons detect chromatin containing HIF-responsive elements (HRE) in regulatory regions of the HIF-2 target genes MMP9 and PAI1. (C) Single ChIP assays in same cells and with same amplicons as in (B), but using antibodies recognizing specific acetylation marks in histone 3 induced by Cbp, H3K18ac and H3K27ac, as well as a histone 3 mark not modified by Cbp, H3K9ac. Comparison of samples within a given condition was performed by one-tailed unpaired t test with decreased samples noted (*, P<0.05). Values indicated are means with SD.

We also asked if nuclear localized Acss2 broadly controls epigenetic marks. Global H3K18ac and H3K27ac levels increase in WT, but are unchanged in CYT, Acss2 knockdown/rescue cells (Fig 8A). Epigenetic marks induced by other modifying enzymes (H3K9ac, H3K14ac, H3R17me2), poised enhancers (H3K9me3, H3K27me3), or histone 3 (pan histone3) levels are grossly unchanged in WT or CYT Acss2 knockdown/rescue cells (Fig 8A and S5 Fig).

Consistent with WT Acss2 being required for stable Cbp/HIF-2α complex formation, efficient recruitment of endogenous HIF-2α to promoter regions of HIF-2 target genes (MMP9, PAI1, VEGFa, GLUT1), as assessed by single ChIP after either hypoxia or low glucose exposure, is evident when wild-type (WT), but not cytosol-restricted (CYT), Acss2 is expressed (Fig 8A and S6A Fig). Furthermore, sequential ChIP reveals that endogenous Cbp is also recruited with endogenous HIF-2α when WT, but not CYT, Acss2 is expressed (Fig 8A and S6A Fig).

We asked if disrupting Cbp/HIF-2α interactions, through elimination of nuclear localizing Acss2, also perturbs histone marks at HIF-2 target gene regulatory regions. H3K18ac and H3K27ac epigenetic marks at regulatory regions of HIF-2 target genes (MMP9, PAI1, VEGFa, GLUT1) increase during stress in WT Acss2 knockdown/rescue cells, but are reduced in CYT Acss2 knockdown/rescue cells (Fig 8B and S6B Fig). These epigenetic marks are not decreased in a HIF-1 regulatory region (PGK1) or a non-HIF promoter (RPL13A) for CYT Acss2 knockdown/rescue cells (S6B Fig).

### Acetylated HIF-2α regulates *in vitro* tumor cell properties

**Fig 9:**
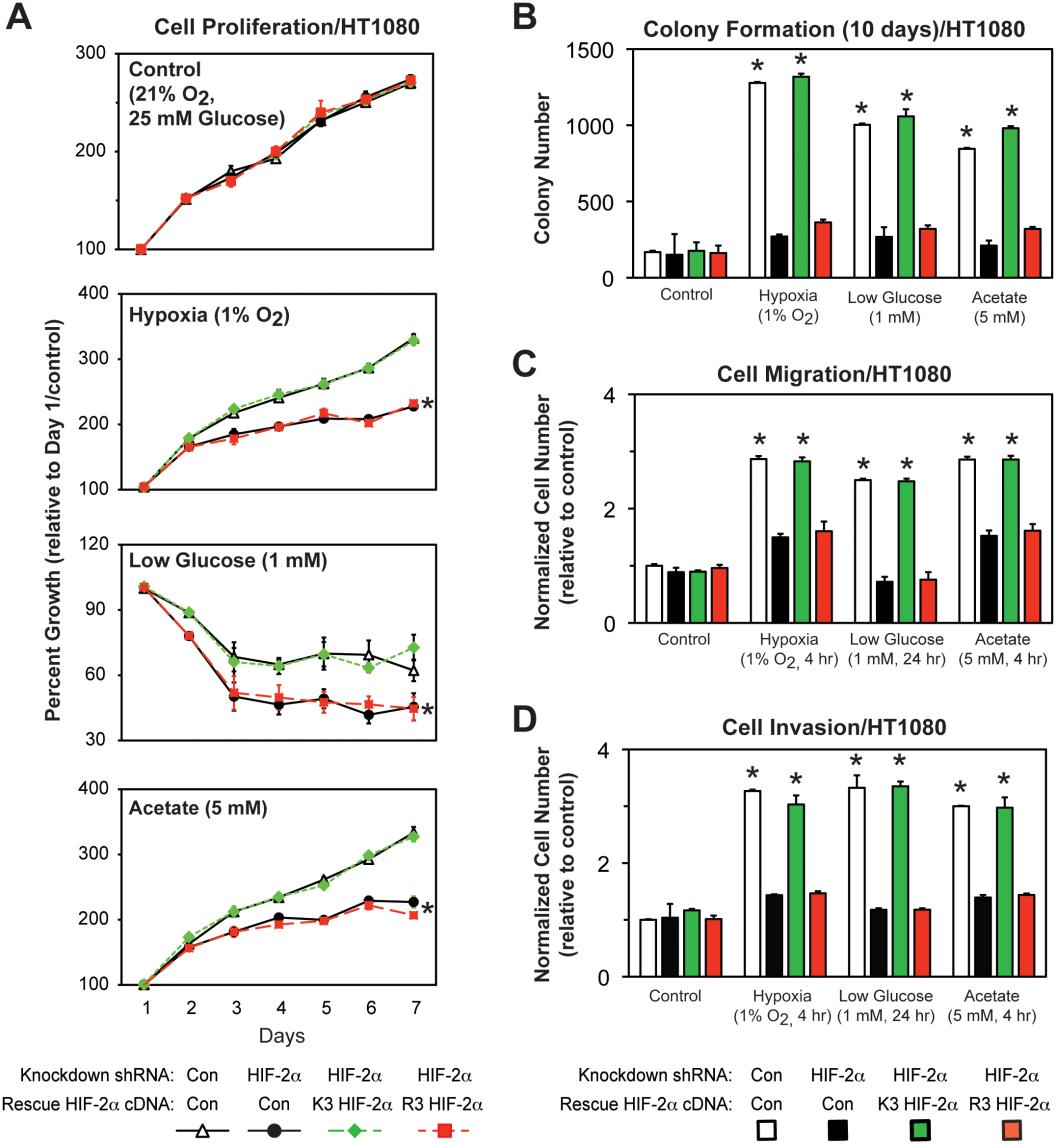
Acetylated HIF-2α regulates *in vitro* tumor cell properties. (A) Cell proliferation of stably transformed control or HIF-2α shRNA knockdown HT1080 cells, or stably transformed knockdown/rescue HT1080 cells expressing wild-type (K3) or arginine-lysine substituted mutant (R3) HIF-2α. Cells were exposed to control (21% O_2_, 25 mM glucose), hypoxic (1% O_2_), or low glucose (1 mM) conditions, or were supplemented with acetate (5 mM) (n = 8/treatment/day). (B) Colony formation, (C) Cell migration, or (D) Cell invasion after ten days of the same cells assessed under identical conditions (n = 3/treatment). Comparison of samples within a given condition on the day of interest was performed by two-way (cell proliferation) or one-way (colony formation, cell migration, cell invasion) ANOVA followed by Dunnett’s multiple comparisons test using control shRNA knockdown/control rescue as reference with decreased samples noted (*, P<0.05). For proliferation data, the indicated samples (*) differ from control cells for at least five of the last six days in this seven-day protocol. Other samples have no or one difference during this same period. Values indicated are means with SD.

Specific HIF-2α lysine residues are required for stable Cbp/HIF-2α complex formation (Fig 1), induction of HIF-2 target genes (Fig 5), and chromatin remodeling (Fig 7) in cells exposed to hypoxia or glucose deprivation. To assess whether HIF-2α acetylation is required for maintenance of *in vitro* tumor cell properties, stably transformed knockdown/rescue HT1080 cells expressing ectopic K3 or R3 HIF-2α were examined. Under stress conditions (hypoxia or low glucose exposure) or following acetate supplementation, R3 HIF-2α was impaired in its ability to rescue cell proliferation, colony formation, cell migration, and cell invasion, tumor cell processes also blunted by endogenous HIF-2α knockdown (Fig 9A–9D).

### Nuclear Acss2 regulates *in vitro* tumor cell properties

**Fig 10:**
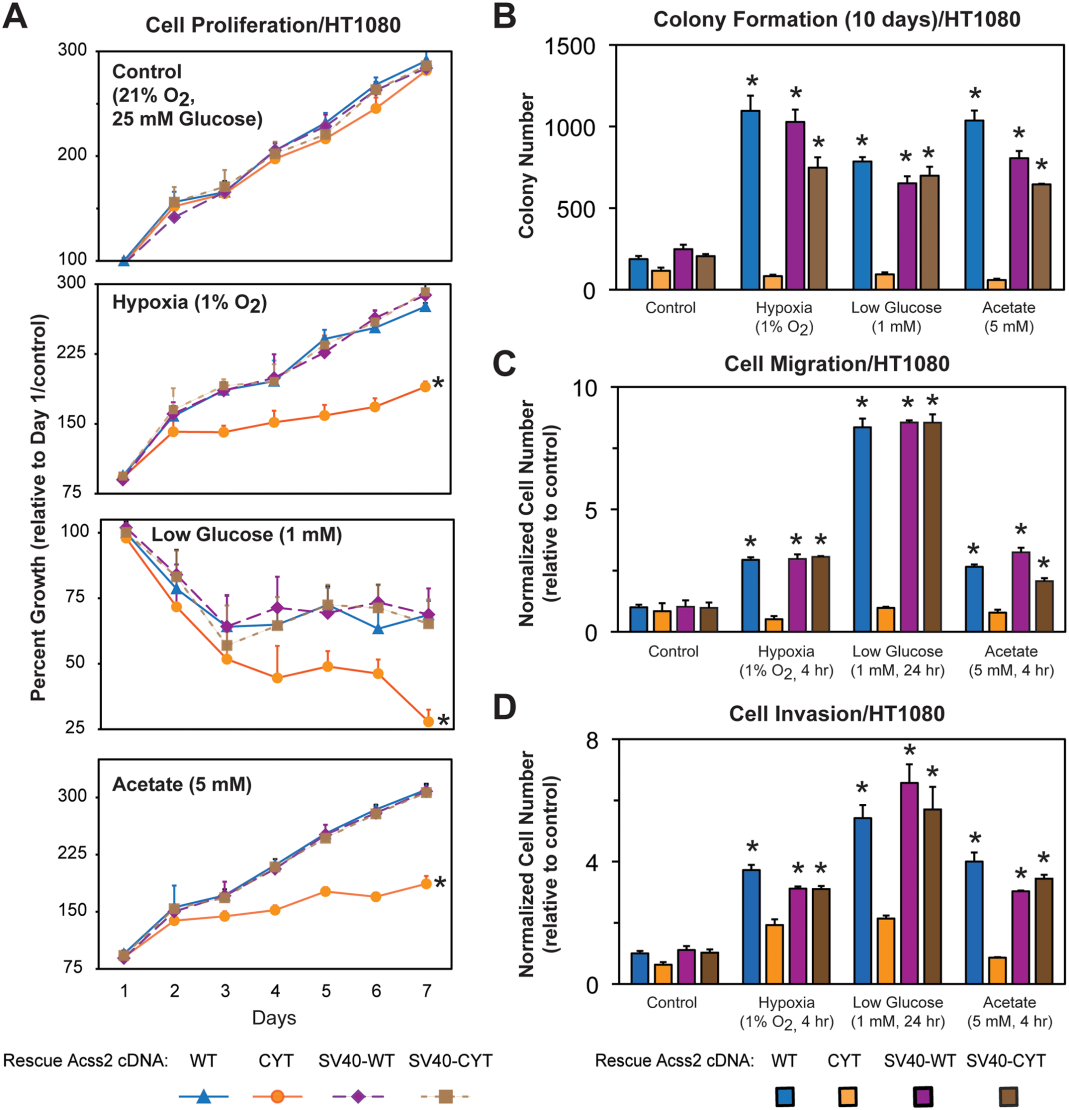
Nuclear Acss2 regulates *in vitro* tumor cell properties. (A) Cell proliferation of stably transformed knockdown/rescue HT1080 cells expressing wild-type (WT) Acss2, cytosol-restricted mutant (CYT) Acss2, or the same two proteins with the SV40 large T antigen nuclear localization signal fused to the amino terminus (SV40-WT, SV40-CYT). Cells were exposed to control, hypoxic, or low glucose conditions, or were supplemented with acetate (5 mM) (n = 8/treatment/day). (B) Colony formation, (C) Cell migration, or (D) Cell invasion after ten days of the same cells assessed under identical conditions (n = 3/treatment). Comparison of samples within a given condition on the day of interest was performed by two-way (cell proliferation) or one-way (colony formation, cell migration, cell invasion) ANOVA followed by Dunnett’s multiple comparisons test using control shRNA knockdown/control rescue as reference with decreased samples noted (*, P<0.05). For proliferation data, the indicated samples (*) differ from control cells for at least five of the last six days in this seven-day protocol. Other samples have no or one difference during this same period. Values indicated are means with SD.

Enzymatically active Acss2, which is present in the nucleus following stress or acetate exposure (Fig 3), is required for HIF-2α acetylation (Fig 4), induction of HIF-2 target genes (Fig 6), and chromatin remodeling (Fig 8) in cells exposed to hypoxia or glucose deprivation. Acss2 is necessary for effective growth in tumor cells[14]. We reasoned that tumor growth and metastasis depends upon Acss2 signaling in the nucleus rather than Acss2 cytosolic lipid synthesis. Therefore, we measured cell survival, migration, invasion, and colony formation of stably transformed HT1080 cells lacking endogenous Acss2 and expressing ectopic WT, CYT, SV40-WT, or SV40-CYT Acss2 under control, hypoxia, low glucose, or acetate supplemented conditions. Only CYT Acss2 is impaired in its ability to rescue tumor cell processes (Fig 10A–10D).

### Acetylated HIF-2α regulates tumor cell growth and metastasis

**Fig 11:**
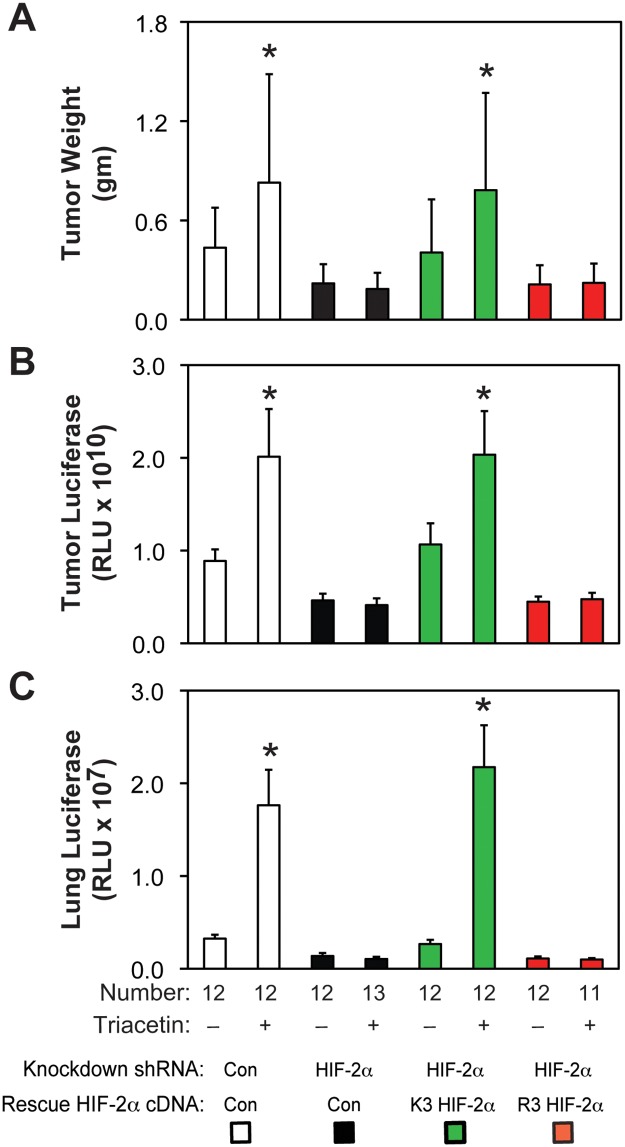
Acetylated HIF-2α regulates tumor cell growth and metastasis. (A) Primary tumor weight as well as (B) luciferase activity and (C) lung metastatic burden assessed by luciferase activity in nude mice carrying flank tumors derived from luciferase-expressing stably transformed control or HIF-2α knockdown HT1080 cells, or stably transformed knockdown/rescue HT1080 cells expressing wild-type (K3) or arginine-lysine substituted mutant (R3) HIF-2α. Luciferase activity was assessed by triplicate measurements of tissue extracts. Mice in each group were treated with control or oral acetate (triacetin) delivered by oral gavage. Comparison within each shRNA knockdown/control rescue pair treated with vehicle or acetate (triacetin) was performed by one-tailed unpaired t test with Welch’s correction for groups of equal sample size or by one-tailed Mann-Whitney test for groups of unequal sample size with reductions indicated (*, P<0.05). Values indicated are means with SD (weights) or SEM (luciferase measurements).

We asked if stably transformed acetylation-deficient HIF-2α knockdown/rescue HT1080 cells have impaired *in vivo* growth and metastatic potential. Primary tumor weight and luciferase activity as well as metastatic lung luciferase activity are similar in mice with control shRNA knockdown or acetylation-intact (K3) HIF-2α knockdown/rescue flank tumors (Fig 11A–11C). In contrast, mice with flank tumors derived from HIF-2α shRNA knockdown or acetylation-defective (R3) HIF-2α knockdown/rescue cells have blunted primary tumor weight and luciferase activity as well as metastatic lung tumor activity. Triacetin, an oral acetate compound, augments growth and metastasis of control shRNA knockdown or K3 HIF-2α knockdown/rescue cells, but not HIF-2α shRNA knockdown or R3 HIF-2α knockdown/rescue cells.

### Nuclear Acss2 regulates tumor cell growth and metastasis

**Fig 12:**
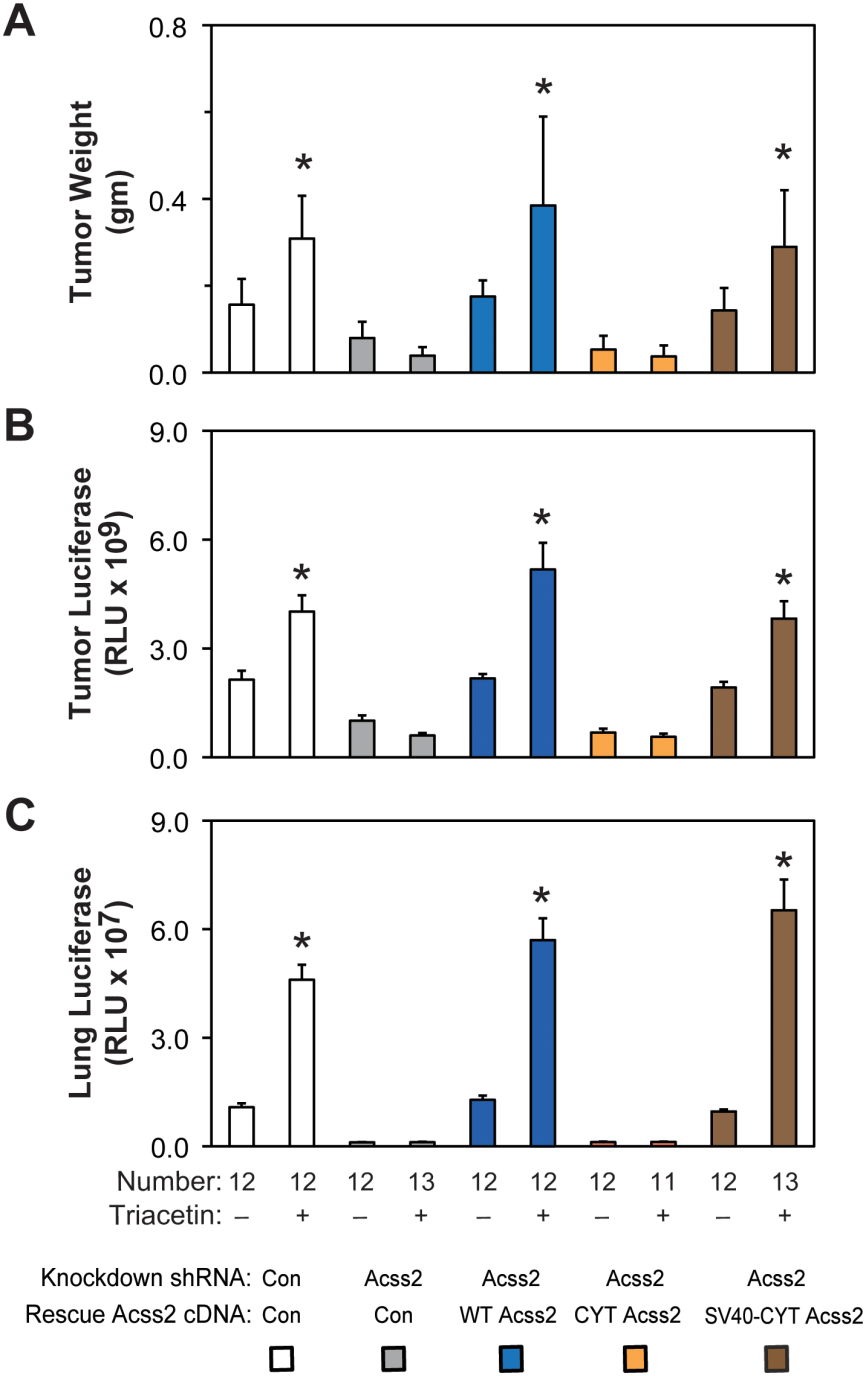
Nuclear Acss2 regulates tumor cell growth and metastasis. (A) Primary tumor weight as well as (B) luciferase activity and (C) lung metastatic burden assessed by luciferase activity in nude mice carrying flank tumors derived from luciferase-expressing stably transformed control or Acss2 knockdown HT1080 cells, or stably transformed knockdown/rescue HT1080 cells expressing wild-type (WT) Acss2, cytosol-restricted mutant (CYT) Acss2, or CYT Acss2 with the SV40 large T antigen nuclear localization signal fused to the amino terminus (SV40-CYT). Luciferase activity was assessed by triplicate measurements of tissue extracts. Mice in each group were treated with control or oral acetate (triacetin) delivered by oral gavage. Comparison within each shRNA knockdown/control rescue pair treated with vehicle or acetate (triacetin) was performed by one-tailed unpaired t test with Welch’s correction for groups of equal sample size or by one-tailed Mann-Whitney test for groups of unequal sample size with reductions indicated (*, P<0.05). Values indicated are means with SD (weights) or SEM (luciferase measurements).

Next, we asked if cytosolic restricted Acss2 knockdown/rescue HT1080 cells have impaired *in vivo* growth and metastatic potential. Primary tumor weight and luciferase activity as well as metastatic lung luciferase activity are similar in mice with control shRNA knockdown, WT Acss2 knockdown/rescue, or SV40-CYT Acss2 knockdown/rescue flank tumors (Fig 12A–12C). In contrast, mice with flank tumors derived from Acss2 shRNA knockdown or CYT Acss2 knockdown/rescue cells have blunted primary tumor weight and luciferase activity as well as metastatic lung tumor activity. Triacetin augments growth and metastasis of control shRNA knockdown, WT Acss2 knockdown/rescue, or SV40-CYT Acss2 knockdown/rescue cells, but not of Acss2 shRNA knockdown or CYT Acss2 knockdown/rescue cells.

## Discussion

**Fig 13:**
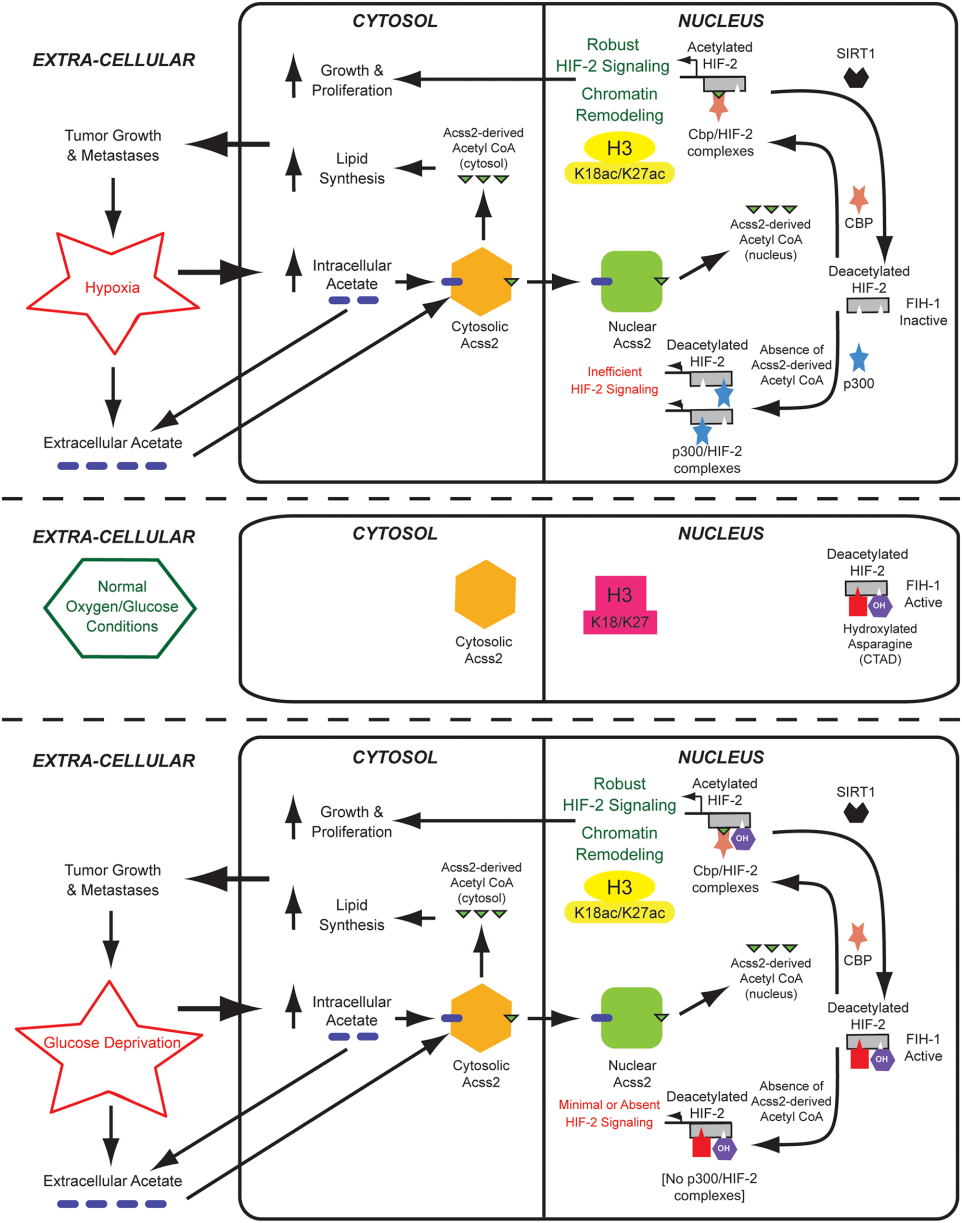
The Acss2/Cbp/Sirt1/HIF-2 axis regulates tumor cell growth and metastasis. An acetate switch controls Cbp/HIF-2α interactions during stress, which is activated by increased endogenous acetate generated in response to stress. Oral acetate also activates the acetate switch to stimulate tumor cell function, which potentially links tumor growth and metastasis with nutritional cues. Nuclear-localized Acss2 is the molecular mediator of the acetate switch and supplies Cbp with a specific pool of nuclear acetyl CoA used in the acetylation of HIF-2α. Cbp and HIF-2α act in concert as a downstream effector of the acetate switch to regulate genetic as well as epigenetic events. Sirt1 regenerates deacetylated HIF-2α, which undergoes repetitive acetylation as long as the nuclear acetyl CoA pool from Acss2 is present.

Although described in lower organisms [26] and postulated in higher metazoans [27], an acetate switch in mammals had eluded detection. Acetate increases during oxygen or glucose deprivation, is converted into acetyl CoA by acetate-dependent acetyl CoA synthetases [27]. We recently reported that a mammalian acetate switch plays a context-dependent role in stress signaling by one HIF family member, HIF-2 [14, 16]. We propose that Acss2, similar to its prokaryotic homologue Acs [26, 28] or yeast homologue Acss2p [29, 30], functions as an acetate-activated switch to alter gene expression patterns, which includes *de novo* genetic (transcriptional) as well as epigenetic (altered histone marks) events (Fig 13).

The acetate-dependent acetyl CoA generator Acss2 was initially investigated as a cytosolic acetyl CoA generator for lipid biosynthesis [31]. Indeed, Acss2 knockdown substantially reduces acetate-derived lipid synthesis as well as flank tumor growth [14], consistent with previous observations implicating Acss2 in this process [32]. However, the molecular basis for impaired growth of tumors bearing mutant Acss2 is not primarily due to effects on anabolic processes. The cytosol-restricted Acss2 examined in this study retains lipid synthesis capacity, yet is markedly impaired in tumor cell function, indicating Acss2 possesses other properties that regulate tumor growth and metastasis.

Instead of existing in equilibrium [33], cytosolic and nuclear acetyl CoA pools in eukaryotic cells are likely sequestered [29, 30]. We hypothesize that dynamic changes in nuclear pools of acetyl CoA, the other substrate beside HIF-2α lysines in the Cbp-mediated HIF-2α acetylation reaction, regulate HIF-2α acetylation, Cbp/HIF-2α complex formation, and hence HIF-2 signaling. This signaling function of Acss2 requires that it transit from the cytosol to the nucleus. However, nuclear localization of Acss2—as occurs with hypoxia, low glucose, or acetate exposure—is necessary, but not sufficient, to augment HIF-2 signaling. Increased acetate levels that follow stress exposure trigger Acss2 nuclear translocation and acetyl CoA production.

The mutations used to generate a cytosol-restricted Acss2 are natural sequence variants found in a prokaryotic acetyl CoA synthetase (ACS) [34]. Although nuclear translocation is not relevant for prokaryotic ACS proteins, eukaryotic Acss2 stress signaling share some commonalities with prokaryotic ACS stress signaling. Acetate in *E*. *coli* increases during hypoxia [35] and is used by ACS to generate acetyl CoA [26]; acetate in eukaryotic cells increases in response to hypoxia (or glucose deprivation) and is likewise used by Acss2 to generate acetyl CoA [14, 16]. Sir2, a stress-responsive genetic regulator, modulates ACS activity [36]; Sirt1, the eukaryotic Sir2 homologue, also may play a putative role in regulation of Acss2 activity. However, there are some differences in acetate and hypoxia stress signaling in prokaryotes and eukaryotes. Acetate signaling by ACS is controlled by dynamic acetate-responsive acetylation/deacetylation modifications [37]. It is not known whether Acss2 is regulated in a likewise manner. Hypoxia sensing by ArcA, a prokaryotic functional homologue of HIF-1, is activated by limiting oxygen states[38], but operates independent of ACS [39, 40]. In eukaryotes, acetate and hypoxia signaling are linked through the actions of Acss2, Cbp, and HIF-2.

Since the initial reports of Acss2 in stress signaling [14, 16], additional studies have expanded our knowledge of the proposed role that acetyl CoA, acetate, and Acss2 play in epigenetic regulation during metabolic stress states [41–43]. Consistent with its earlier reported role as a biochemical flare [14, 16], acetate increases in hypoxic cancer cells and induces hyper-acetylation of histone 3 in a partially Acss2-dependent manner [44]. We confirm these findings with respect to effects of Acss2 depletion on histone acetylation, although we note that loss of Acss2 results in global depletion of H3K27, but not H3K9, in addition to global depletion of H3K18 acetylation marks. We propose a mechanism whereby HIF-2 directly recruits Cbp to hypoxia-induced target genes, which facilitates Cbp acetylation of histone 3 at these target genes.

Acss2-mediated acetyl CoA generation in both the cytosol and nucleus are important in cancer cells. Reduced levels of Acss2 result in acetyl CoA depletion, protein deacetylation, and autophagy with the latter likely a result of loss of cytosolic acetyl CoA. However, the impact of Acss2 knockdown on protein acetylation is not likely due to reduced diffusion of acetyl CoA from the cytosol to the nucleus. We find that Acss2-generated acetyl CoA is used by Cbp in acetylation of HIF-2α as well as histone 3, but only if Acss2 generates a specific nuclear acetyl CoA pool in the nucleus under stress. While this manuscript was in preparation, a study also reported that Acss2 nuclear translocation is required for histone 3 acetylation, specifically at lysosomal and autophagosomal gene promoter regions, during glucose deprivation [45]. However, we find that this stress-induced, nuclear Acss2-dependent acetyl CoA generation and histone acetylation occurs during hypoxia as well as glucose deprivation.

The specific association of Acss2 with Cbp and subsequent effects on histone acetylation also has been shown in a recent study examining the role of Acss2 in neuronal memory [46]. An increase in specific histone 3 acetylation marks, H3K9 and H3K27, occurs during neuronal differentiation, which is associated with a transition from cytosolic to nuclear Acss2 localization. Similar to our results, Acss2 depletion results in a global decrease in acetylated H3K27 levels. In contrast, these investigators also note a decrease in global acetylated H3K9 levels, which may reflect differences in cell types used in the respective study (primary hippocampal neurons versus fibrosarcoma cells). Acetylated H3K18 levels were not assessed in that study. The investigators also noted physical association of Acss2 and Cbp evident by immunoprecipitation studies, which may explain why Acss2 and Cbp are enriched in chromatin from neuronal tissue. Thus, the combined action of Acss2 and Cbp on histone acetylation in specific regulatory regions may be dictated in part by interactions of Acss2 with Cbp, but likely are also due to Acss2-dependent interactions of Cbp with transcriptional regulators such as HIF-2, which require the specific subcellular pool of acetyl CoA generated by Acss2 in the nucleus.

In this study, we show that Acss2 regulates dynamic HIF-2α acetylation, Cbp/HIF-2α complex formation, Cbp/HIF-2 signaling, epigenetic remodeling, and tumor cell growth and metastasis (Fig 13). Ties between intermediary metabolism and cancer biology through signal transduction pathways are becoming increasingly evident [41–43]. The acetate/Acss2 switch acts in concert with HIF-2 during hypoxia and glucose deprivation, stresses that are frequently encountered in solid tumors. Oral acetate substantially affects flank tumor outcomes in an Acss2 and HIF-2 dependent manner. Serum acetate originates from endogenous and exogenous sources, the latter dictated by dietary and gastrointestinal bacterial interactions. Defining the inner workings of the mammalian acetate switch in normal as well as in transformed cells will inform us how intermediary metabolism and stress recognition are exquisitely linked to the prosurvival cellular response. Manipulation of this pathway may define a novel approach for intervening in cancer and possibly other disease states where the acetate switch is relevant.

## Supporting information

S1 Fig CYT Acss2 exhibits impaired Acss2 nuclear translocation during stress. Low-power magnification of ectopic V5-tagged wild-type (WT) or cytosol-restricted mutant (CYT) Acss2 in HT1080 cells under basal and stress conditions revealing subcellular localization by immunofluorescence and merging with Hoechst-stained cells to detect nuclei.
(TIF)

S2 Fig Depletion of Acss2 has no effect on global acetylation. Global acetylation in HT1080 cells depleted of Acss2 by shRNA-mediated knockdown compared to control knockdown cell lines does not differ when maintained under control, hypoxia, or low glucose conditions.
(TIF)

S3 Fig Acetylated HIF-2α does not regulate non-Cbp-regulated stress remodeling. Global epigenetic marks associated with other modifying enzymes (H3R17me2), poised enhancers (H3K9me3, H3K27me3), or histone 3 (pan histone3) levels are grossly unchanged in K3 or R3 HIF-2α knockdown/rescue cells maintained under control, hypoxia, or low glucose conditions.
(TIF)

S4 Fig Acetylated HIF-2α regulates Cbp-mediated stress remodeling. Single and sequential chromatin immunoprecipitation (ChIP) assays in stably transformed HT1080 knockdown/rescue cells expressing ectopic V5-tagged K3 or R3 HIF-2α maintained under control (Con), hypoxia (Hyp), or low glucose (LG) conditions. The single and first stage of the sequential ChIP was performed with antibodies recognizing V5. The second stage of the sequential ChIP was performed with antibodies recognizing endogenous Cbp. The amplicons detect chromatin containing HIF-responsive elements (HRE) in regulatory regions of the HIF-2 target genes VEGFa and GLUT1. (B) Single ChIP assays in same cells and with same amplicons as in (A) as well as with amplicons recognizing the HIF-1 selective target gene PGK1 and a non-HIF regulated gene RPL13A, but using antibodies recognizing specific marks in histone 3 proteins acetylated by Cbp, H3K18ac and H3K27ac, as well as a histone 3 mark not modified by Cbp, H3K9ac. Comparison of samples within a given condition was performed by one-tailed unpaired t test with significantly decreased samples noted (*, P<0.05). Values indicated are means with SD.
(TIF)

S5 Fig Nuclear Acss2 does not regulate non-Cbp-regulated stress remodeling. Global epigenetic marks associated with other modifying enzymes (H3R17me2), poised enhancers (H3K9me3, H3K27me3), or histone 3 (pan histone3) levels are grossly unchanged in WT or CYT Acss2 knockdown/rescue cells maintained under control, hypoxia, or low glucose conditions.
(TIF)

S6 Fig Nuclear Acss2 regulates Cbp-mediated stress remodeling. Single and sequential chromatin immunoprecipitation (ChIP) assays in stably transformed HT1080 cells expressing ectopic wild-type (WT) or cytosol-restricted (CYT) mutant Acss2 protein maintained under control (Con), hypoxia (Hyp), or low glucose (LG) conditions. The single and first stage of the sequential ChIP was performed with antibodies recognizing endogenous HIF-2α. The second stage of the sequential ChIP was performed with antibodies recognizing endogenous Cbp. The amplicons detect chromatin containing HIF-responsive elements (HRE) in regulatory regions of the HIF-2 target genes VEGFa and GLUT1. (B) Single ChIP assays in same cells and with same amplicons as in (A) as well as with amplicons recognizing the HIF-1 selective target gene PGK1 and a non-HIF regulated gene RPL13A, but using antibodies recognizing specific marks in histone 3 proteins acetylated by Cbp, H3K18ac and H3K27ac, as well as a histone 3 mark not modified by Cbp, H3K9ac. Comparison of samples within a given condition was performed by one-tailed unpaired t test with significantly decreased samples noted (*, P<0.05; **, P<0.10). Values indicated are means with SD.
(TIF)

S1 File Annotated data. An Excel file containing raw data and annotations for all material presented in this study.
(XLSX)
