# Supplementary figures and images for: Coordinate regulation of stress signaling and epigenetic events by Acss2 and HIF-2 in cancer cells
Source: PLoS One. 2017 Dec 27;12(12):e0190241. doi: 10.1371/journal.pone.0190241 (PMC5744998; doi:10.1371/journal.pone.0190241)

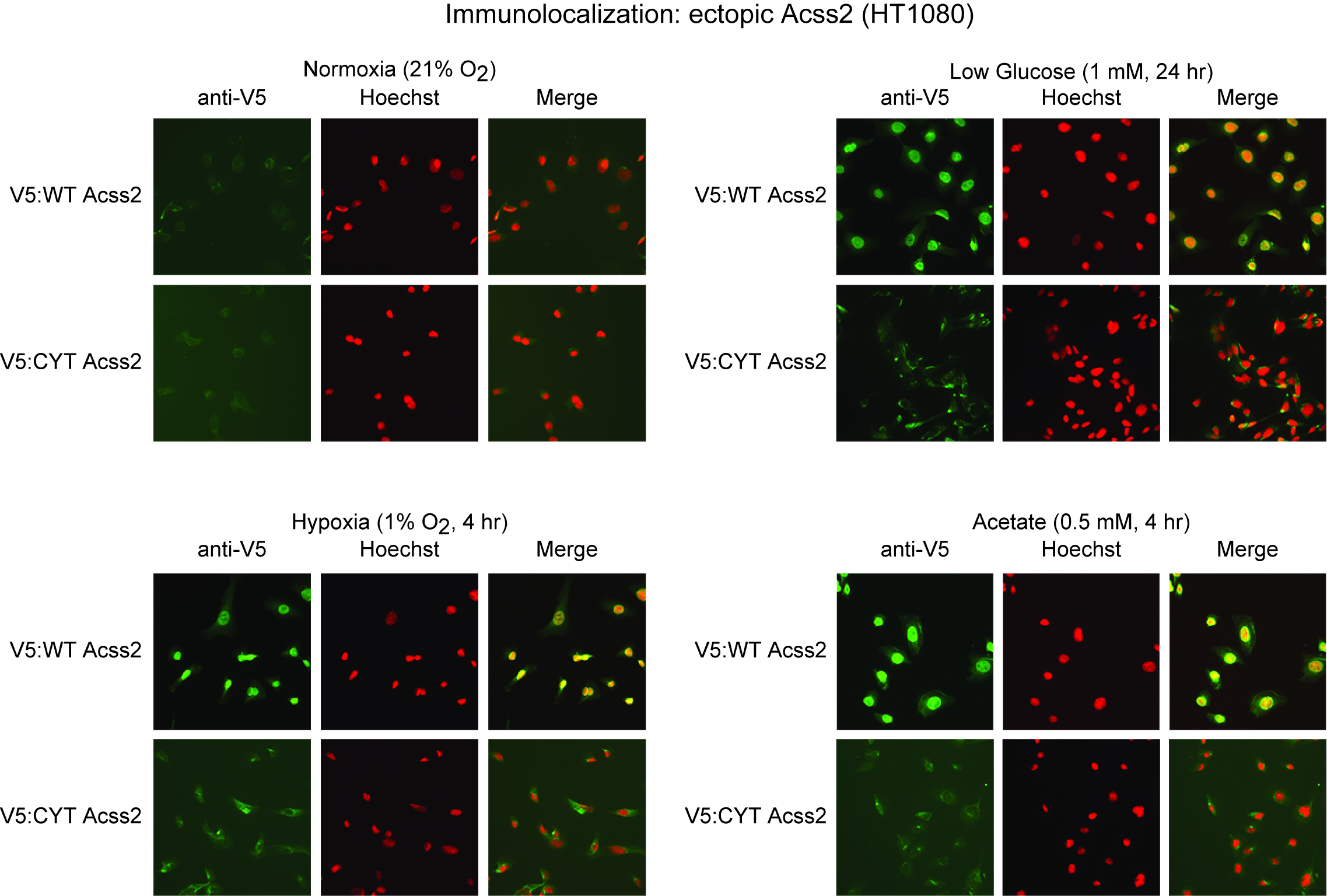

Supplement: S1 Fig — Low-power magnification of ectopic V5-tagged wild-type (WT) or cytosol-restricted mutant (CYT) Acss2 in HT1080 cells under basal and stress conditions revealing subcellular localization by immunofluorescence and merging with Hoechst-stained cells to detect nuclei. (TIF) [file pone.0190241.s001.tif]

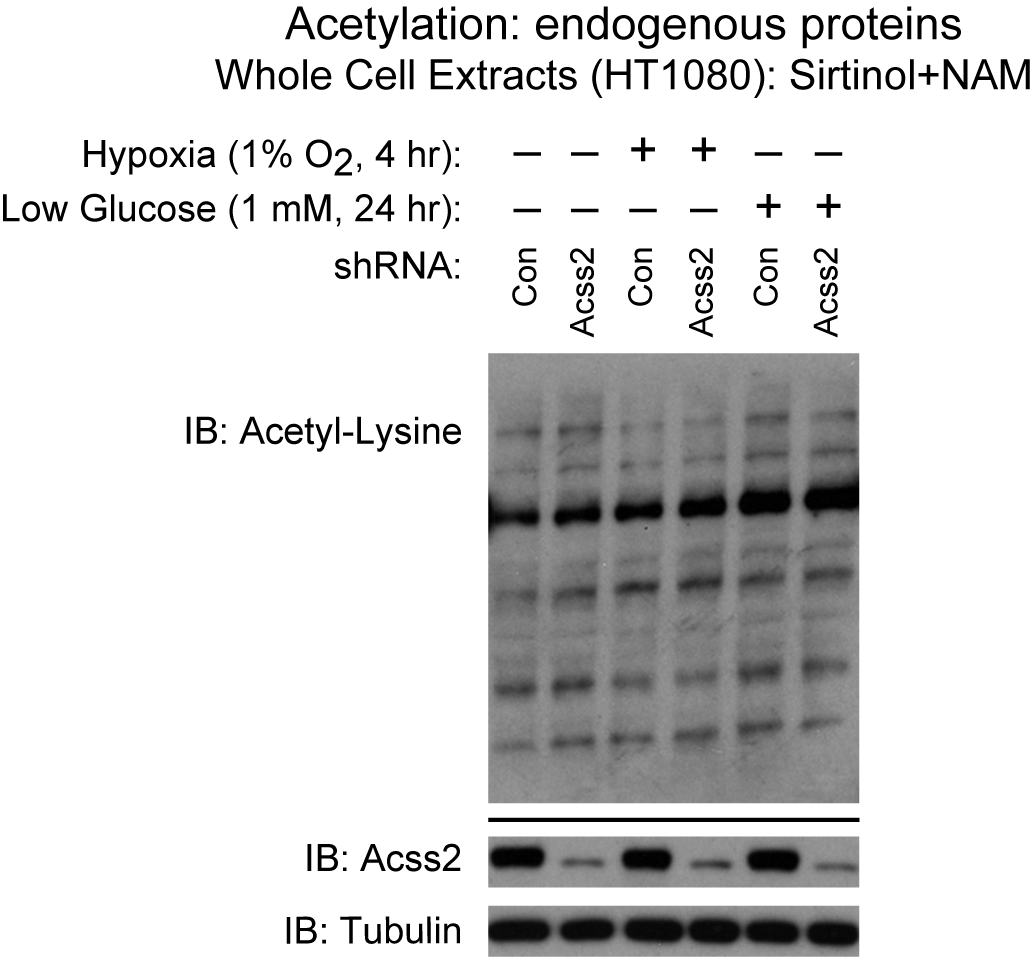

Supplement: S2 Fig — Global acetylation in HT1080 cells depleted of Acss2 by shRNA-mediated knockdown compared to control knockdown cell lines does not differ when maintained under control, hypoxia, or low glucose conditions. (TIF) [file pone.0190241.s002.tif]

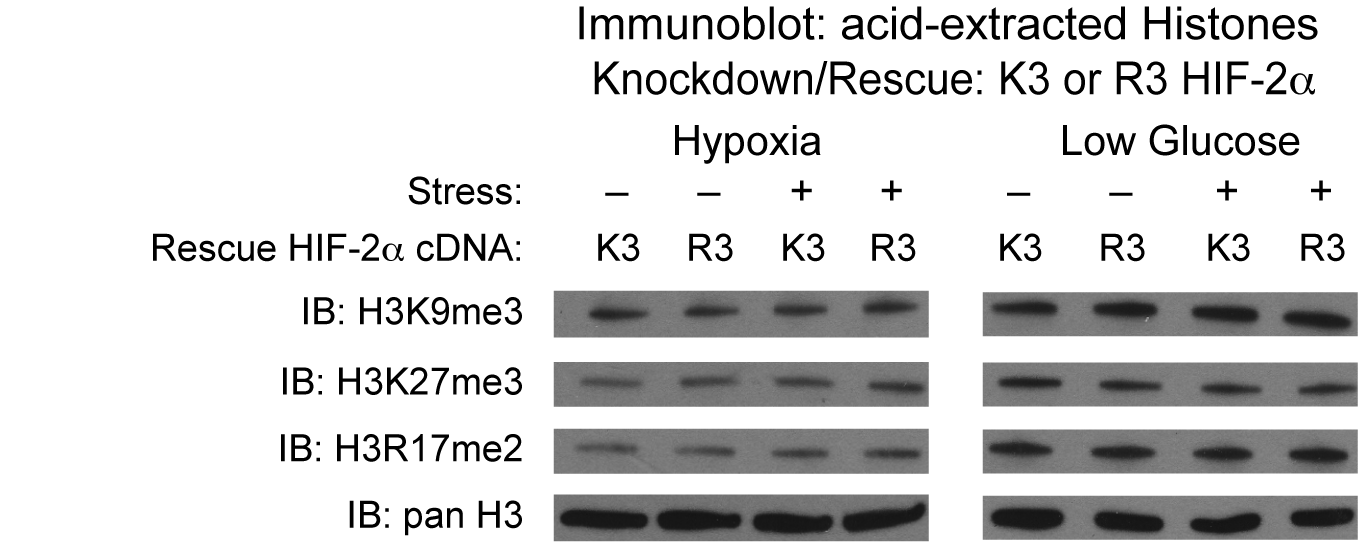

Supplement: S3 Fig — Global epigenetic marks associated with other modifying enzymes (H3R17me2), poised enhancers (H3K9me3, H3K27me3), or histone 3 (pan histone3) levels are grossly unchanged in K3 or R3 HIF-2α knockdown/rescue cells maintained under control, hypoxia, or low glucose conditions. (TIF) [file pone.0190241.s003.tif]

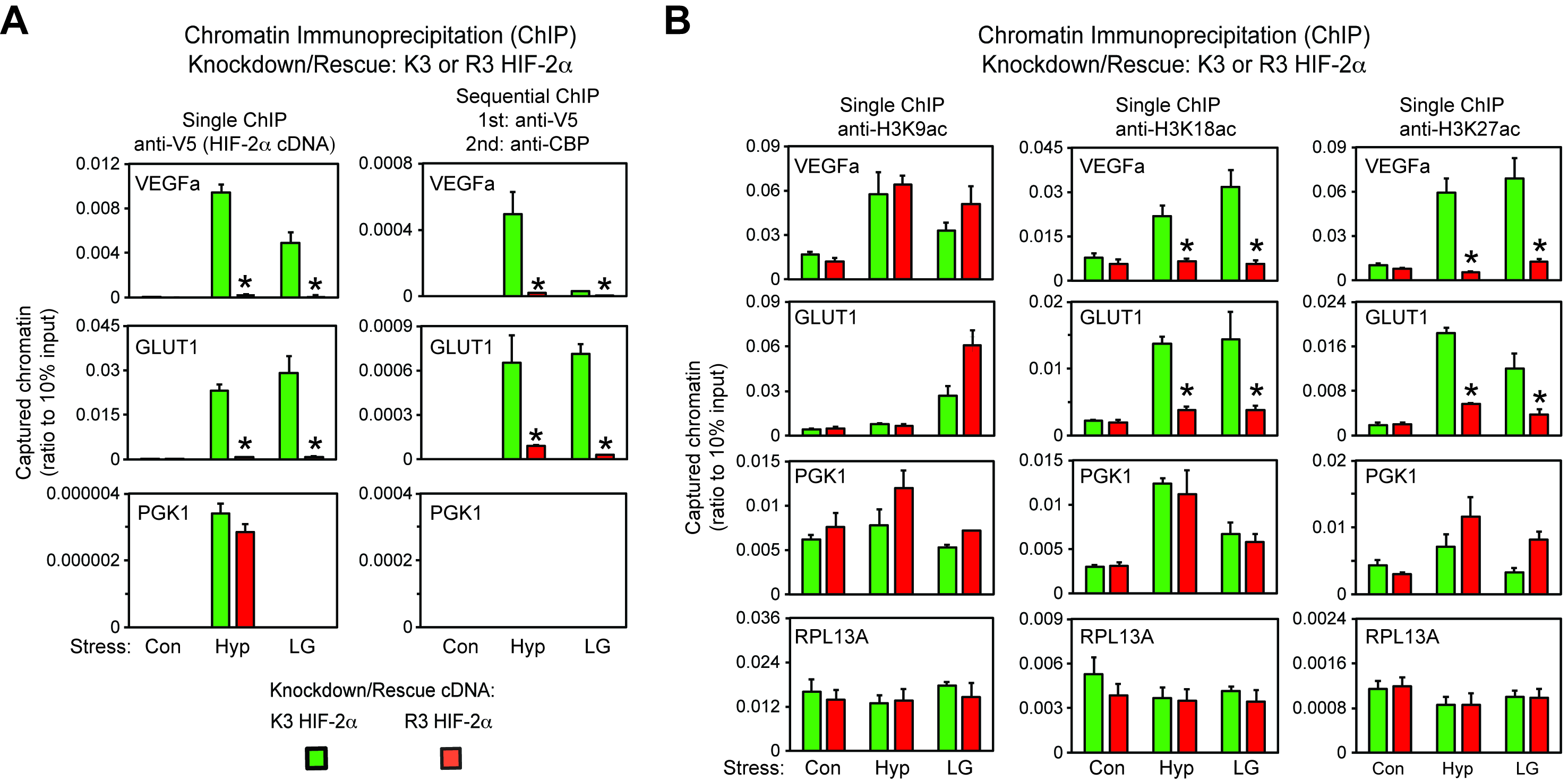

Supplement: S4 Fig — Single and sequential chromatin immunoprecipitation (ChIP) assays in stably transformed HT1080 knockdown/rescue cells expressing ectopic V5-tagged K3 or R3 HIF-2α maintained under control (Con), hypoxia (Hyp), or low glucose (LG) conditions. The single and first stage of the sequential ChIP was performed with antibodies recognizing V5. The second stage of the sequential ChIP was performed with antibodies recognizing endogenous Cbp. The amplicons detect chromatin containing HIF-responsive elements (HRE) in regulatory regions of the HIF-2 target genes VEGFa and GLUT1. (B) Single ChIP assays in same cells and with same amplicons as in (A) as well as with amplicons recognizing the HIF-1 selective target gene PGK1 and a non-HIF regulated gene RPL13A, but using antibodies recognizing specific marks in histone 3 proteins acetylated by Cbp, H3K18ac and H3K27ac, as well as a histone 3 mark not modified by Cbp, H3K9ac. Comparison of samples within a given condition was performed by one-tailed unpaired t test with significantly decreased samples noted (*, P<0.05). Values indicated are means with SD. (TIF) [file pone.0190241.s004.tif]

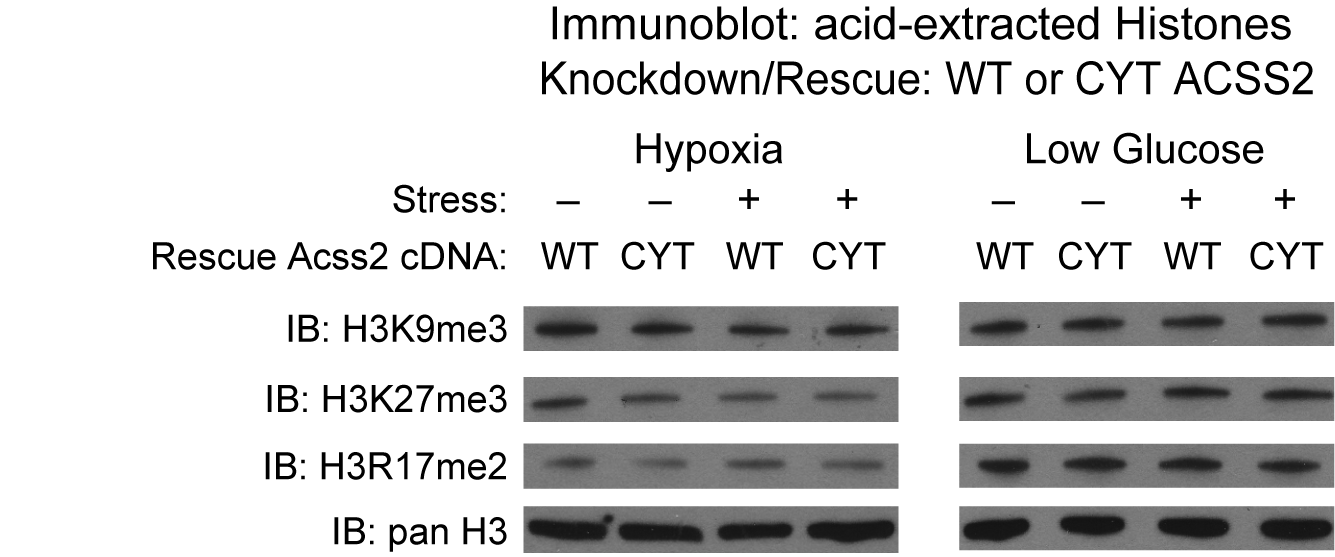

Supplement: S5 Fig — Global epigenetic marks associated with other modifying enzymes (H3R17me2), poised enhancers (H3K9me3, H3K27me3), or histone 3 (pan histone3) levels are grossly unchanged in WT or CYT Acss2 knockdown/rescue cells maintained under control, hypoxia, or low glucose conditions. (TIF) [file pone.0190241.s005.tif]

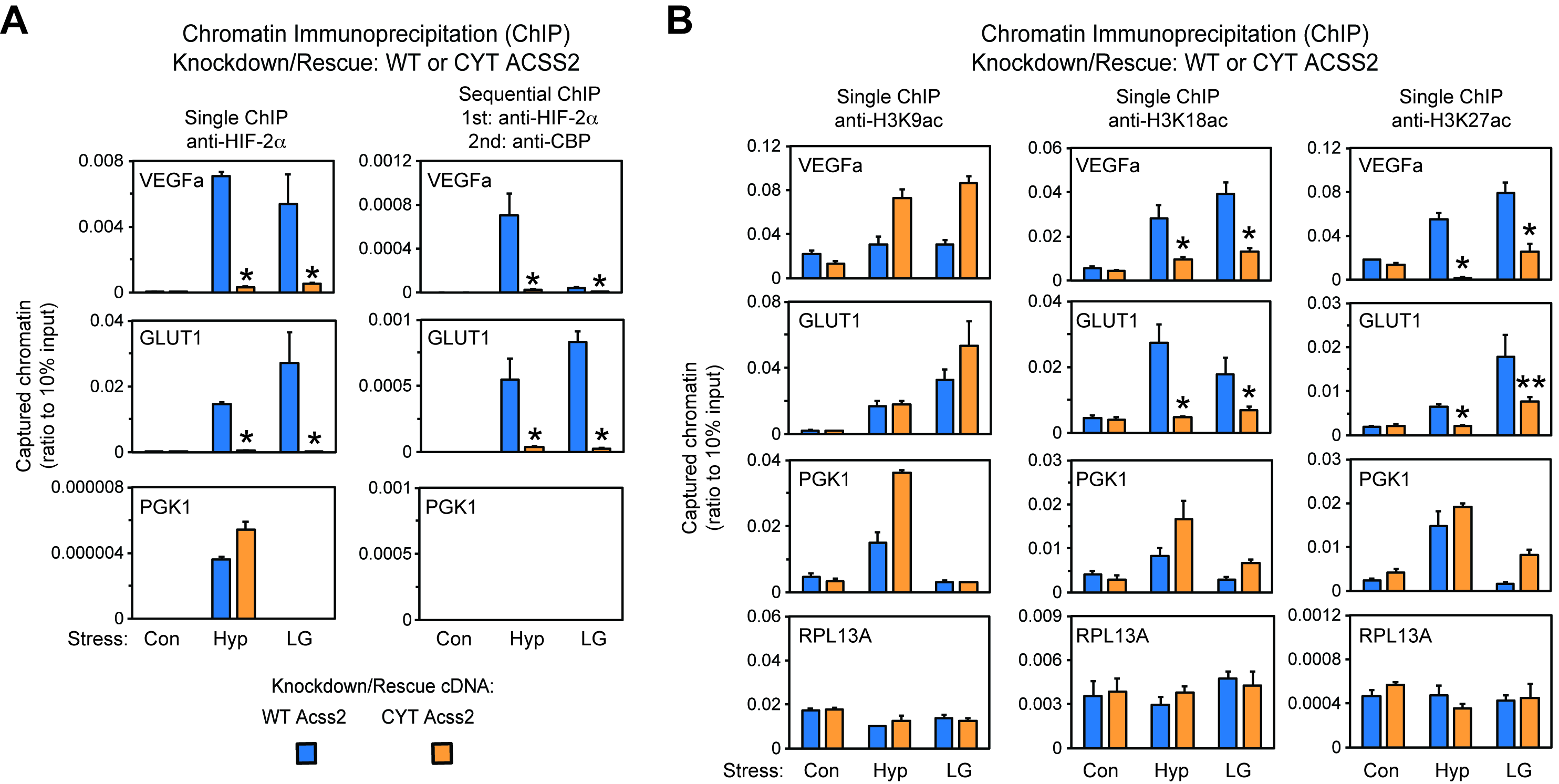

Supplement: S6 Fig — Single and sequential chromatin immunoprecipitation (ChIP) assays in stably transformed HT1080 cells expressing ectopic wild-type (WT) or cytosol-restricted (CYT) mutant Acss2 protein maintained under control (Con), hypoxia (Hyp), or low glucose (LG) conditions. The single and first stage of the sequential ChIP was performed with antibodies recognizing endogenous HIF-2α. The second stage of the sequential ChIP was performed with antibodies recognizing endogenous Cbp. The amplicons detect chromatin containing HIF-responsive elements (HRE) in regulatory regions of the HIF-2 target genes VEGFa and GLUT1. (B) Single ChIP assays in same cells and with same amplicons as in (A) as well as with amplicons recognizing the HIF-1 selective target gene PGK1 and a non-HIF regulated gene RPL13A, but using antibodies recognizing specific marks in histone 3 proteins acetylated by Cbp, H3K18ac and H3K27ac, as well as a histone 3 mark not modified by Cbp, H3K9ac. Comparison of samples within a given condition was performed by one-tailed unpaired t test with significantly decreased samples noted (*, P<0.05; **, P<0.10). Values indicated are means with SD. (TIF) [file pone.0190241.s006.tif]
